# Elevated Levels of an Enzyme Involved in Coenzyme B_12_ Biosynthesis Kills Escherichia coli

**DOI:** 10.1128/mbio.02697-21

**Published:** 2022-01-11

**Authors:** Victoria L. Jeter, Jorge C. Escalante-Semerena

**Affiliations:** a Department of Microbiology, University of Georgia, Athens, Georgia, USA; University of Kent; University of Michigan—Ann Arbor

**Keywords:** coenzyme B_12_ biosynthesis, proton motive force, cell membrane metabolism, cell membrane stability, cell death, cobamide 5′-phosphate synthase

## Abstract

Cobamides are cobalt-containing cyclic tetrapyrroles involved in the metabolism of organisms from all domains of life but produced *de novo* only by some bacteria and archaea. The pathway is thought to involve up to 30 enzymes, five of which comprise the so-called “late” steps of cobamide biosynthesis. Two of these reactions activate the corrin ring, one activates the nucleobase, a fourth one condenses activated precursors, and a phosphatase yields the final product of the pathway. The penultimate step is catalyzed by a polytopic integral membrane protein, namely, the cobamide (5′-phosphate) synthase, also known as cobamide synthase. At present, the reason for the association of all putative and bona fide cobamide synthases to cell membranes is unclear and intriguing. Here, we show that, in Escherichia coli, elevated levels of cobamide synthase kill the cell by dissipating the proton motive force and compromising membrane stability. We also show that overproduction of the phosphatase that catalyzes the last step of the pathway or phage shock protein A prevents cell death when the gene encoding cobamide synthase is overexpressed. We propose that in E. coli, and probably all cobamide producers, cobamide synthase anchors a multienzyme complex responsible for the assembly of vitamin B_12_ and other cobamides.

## INTRODUCTION

**FIG 1:**
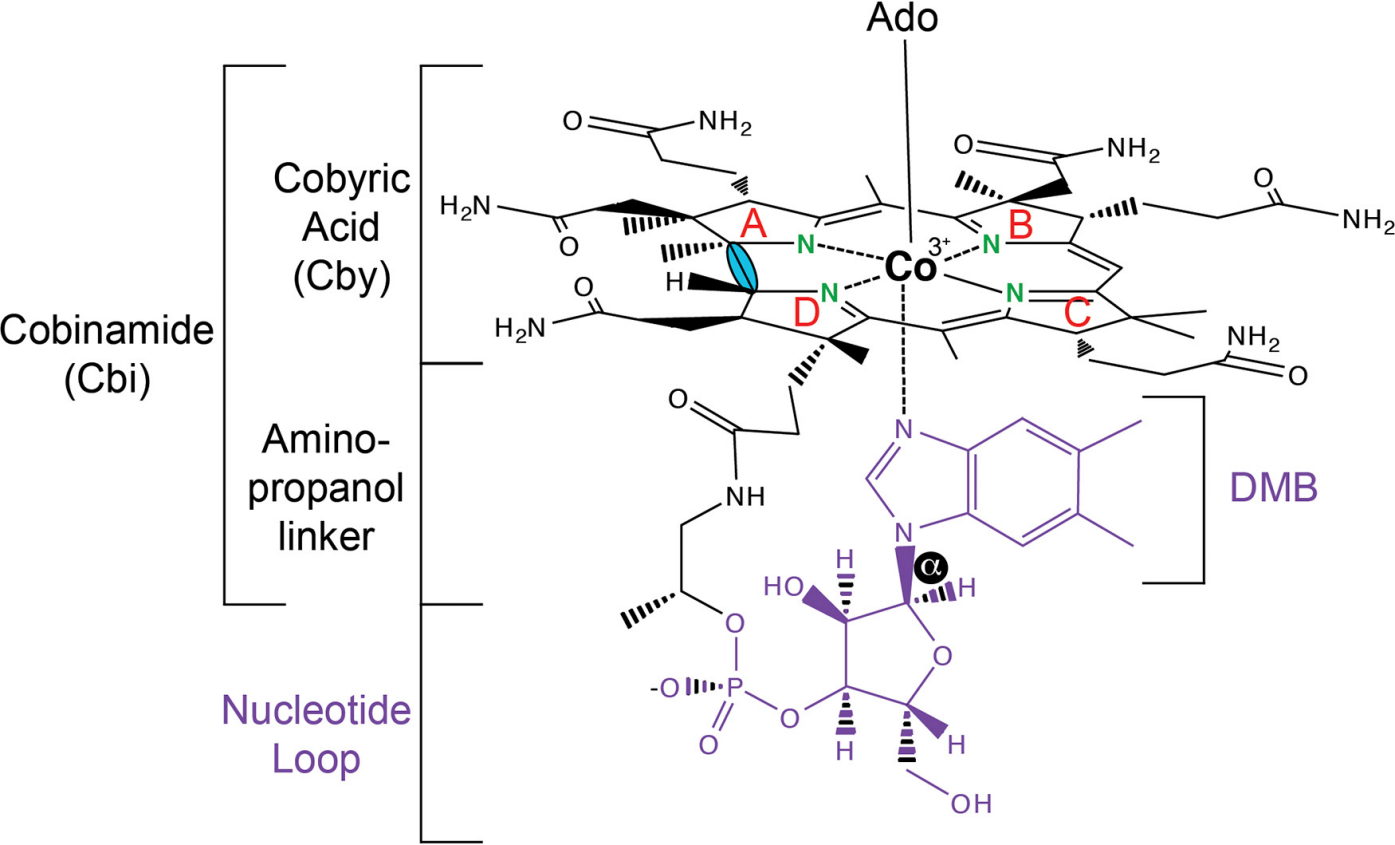
Adenosylcobalamin (AdoCbl; CoB_12_) structure. The chemical structure of AdoCbl, showing the 5′-deoxyadenosine (Ado) upper ligand and the nucleobase 5,6-dimethybenzimidazole (DMB) lower ligand in the ON position. The nucleotide loop is shown in purple, with the α-*N*-glycosidic bond with DMB highlighted in a black background. The Co ion is equatorially coordinated by the N atoms (in green) of the imidazole moieties (A through D in red). Finally, the missing methine group of the corrin ring between rings A and D is highlighted in blue.

Cobamides (Cbas) belong to the family of metal-containing cofactors referred to as “the pigments of life.” Other members of this family of cofactors include hemes, chlorophylls, and coenzyme F_430_ (1, 2). Cbas are cobalt-containing cyclic tetrapyrroles, but they are structurally distinct. Cbas are defined by a cobalt ion that is equatorially coordinated by the imidazole nitrogens of the ring. In addition, Cbas have upper and lower axial ligands, the methine group between rings A and D of the ring is missing, the nucleotide and the ring are attached to each other by a phosphodiester bond, and the *N-*glycosidic bond of the nucleotide is the alpha configuration (Fig. 1). Notably, cobalamin is the cobamide that contains 5,6-dimethylbenzimidazole (DMB) as its nucleobase, and the unpaired electrons of a nitrogen of the imidazole ring of DMB can form a coordination bond with the Co ion of the ring (“base ON” position) or not (“base OFF” position). Finally, the coenzymic form of cobalamin contains a 5′-deoxyadenosine (Ado) group as the upper ligand, and the coenzymic form of cobalamin is also known as adenosylcobalamin (AdoCbl) (Fig. 1) (3–5).

Cbas are required by cells from all domains of life, yet *de novo* synthesis is restricted to some bacteria and archaea (6). Cbas participate in intramolecular rearrangements, reductive dehalogenations, methyl-group transfers, elimination reactions, and radical *S*-adenosylmethionine (SAM)-catalyzed carbon skeleton rearrangements (7). Additionally, Cba-dependent regulation of carotenoid biosynthesis has been shown to use a Cba as a photoreceptor (8). Importantly, Cbas have also been shown to modulate community dynamics (9, 10).

**FIG 2:**
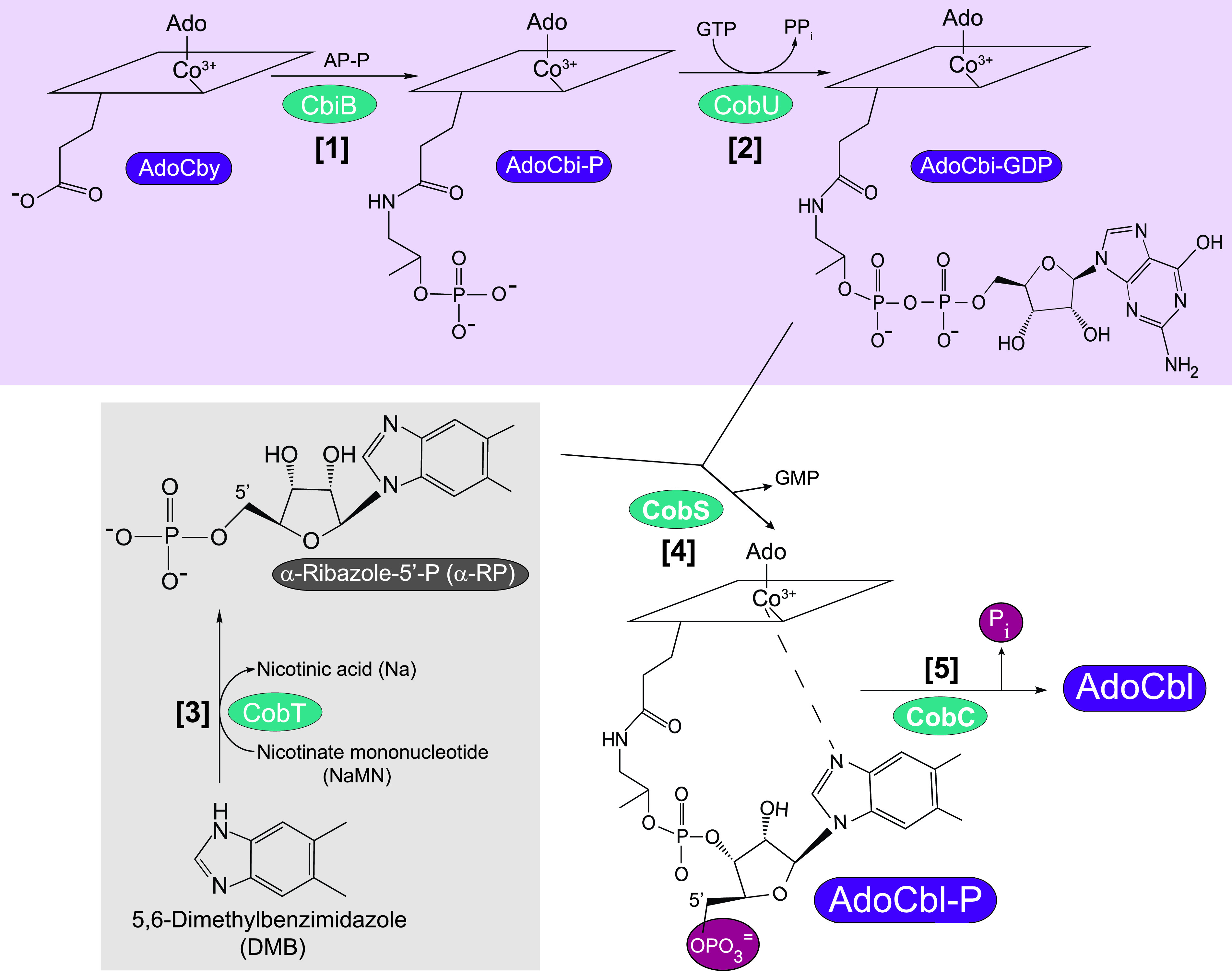
The nucleotide loop assembly (NLA) pathway of S. enterica. The first branch of the pathway (purple background) shows how adenosylcobyric acid (AdoCby) is activated in two steps (labeled [1] and [2]) to yield AdoCbi-GDP. Steps [1] and [2] are catalyzed by the AdoCbi-P synthase (CbiB) and the bifunctional NTP:AdoCbi kinase/NTP/AdoCbi-P guanylyltransferase (CobU) enyzmes, respectively. Nucleobase activation (step [3]; highlighted with a gray background) is catalyzed by the NaMN:base ribosyltransferase (CobT) enzyme. The last two steps ([4] and [5]) are catalyzed by the cobamide synthase (CobS) enzyme and the AdoCbl-P phosphatase (CobC) enzyme, respectively, to yield the final product, AdoCbl (also known as CoB_12_). The five enzymes involved are highlighted with a teal background. The rhomboid cartoon is meant to represent the corrin ring structure of the molecule.

The AdoCbl biosynthetic pathway has three branches: (i) corrin ring synthesis, (ii) corrin ring adenosylation, and (iii) nucleotide loop assembly. The nucleotide loop assembly (NLA) pathway is comprised of five biosynthetic steps that attach the lower nucleobase to the corrin ring. The NLA pathway is divided into three sub-branches: (i) ring activation, (ii) base activation, and (iii) condensation of activated precursors and dephosphorylation of the condensation intermediate (Fig. 2). Here, we use nomenclature that refers to NLA enzymes present in Escherichia coli and Salmonella enterica (6). In the NLA pathway present in S. enterica, corrin ring activation occurs in two steps, starting with the attachment of 1-amino-propanol phosphate (AP-P) to adenosylcobyric acid (AdoCby), forming adenosylcobinamide-phosphate (AdoCbi-P); the AdoCbi-P synthase enzyme (CbiB; EC 6.3.1.10 [not present in E. coli]) catalyzes this reaction (11). In the next step, AdoCbi-P is guanylylated to yield AdoCbi-GDP, the activated form of the corrin ring; this reaction is catalyzed by the guanylyltransferase activity of the bifunctional NTP:AdoCbi kinase (EC 2.7.7.62)/GTP:AdoCbi-P guanylyltransferase (EC 2.7.1.156) CobU enzyme (12–14). Nucleobase activation is performed by the phosphoribosyltransferase (PRTase) (CobT; EC 2.4.2.21) enzyme, which activates DMB by transferring the phosphoribosyl moiety of nicotinate mononucleotide (NaMN) with inversion of configuration of the *N-*glycosidic bond, yielding α-ribazole-phosphate (α-RP), and releasing nicotinic acid (15–19); α-RP is the activated form of DMB. In the third sub-branch of the pathway, cobamide 5′-P synthase (here cobamide synthase [CobS; EC 2.7.8.26]) condenses AdoCbi-GDP and α-RP (20, 21) to yield AdoCbl-P, which in the last step of the pathway is dephosphorylated by CobC (AdoCbl 5′-phosphate phosphatase; EC 3.1.3.73) to yield AdoCbl (22, 23).

CobS is one of two polytopic, integral membrane proteins of the NLA pathway; the other one is CbiB (AdoCbi-P synthase (11, 20). Remarkably, polytopic homologues of CobS and CbiB are present in genomes of all bacteria and archaea that synthesize Cbas, suggesting that the NLA pathway occurs in the membrane of all archaea and bacteria whose genomes have been sequenced. In previous work, we supported this idea by quantifying cobamide synthase activity in membrane preparations of the methanogenic archaeum Methanobacterium thermoautotrophicum (20).

We recently reported the use of liposomes for the functional analysis of the polytopic cobamide synthase (CobS) enzyme (see Fig. S1 in the supplemental material) (24). Here, we report evidence that, in E. coli, high levels of cobamide synthase dissipate the proton motive force (PMF) and decrease membrane stability, arresting growth and ultimately killing the cell. We also show that the detrimental effects of cobamide synthase are counteracted by coexpression of the *cobC* and *pspA* genes, which encode the phosphatase that catalyzes the last reaction of CoB_12_ biosynthesis, and the phage shock protein A (PspA), respectively. *In vitro* evidence shows that association of the CobC phosphatase with liposomes depends on the presence of CobS in the liposome. We propose that multienzyme complex anchored by CobS and probably CbiB catalyzes the late steps of CoB_12_ biosynthesis.

10.1128/mBio.02697-21.1 FIG S1 Model of the polytopic cobamide synthase (CobS) enzyme of S. enterica. This model was generated based on data using LacZ and PhoA fusions placed at different locations of the protein (20). Download FIG S1, PDF file, 1.2 MB. Copyright © 2022 Jeter and Escalante-Semerena. 2022 Jeter and Escalante-Semerena. https://creativecommons.org/licenses/by/4.0/ This content is distributed under the terms of the Creative Commons Attribution 4.0 International license.

## RESULTS AND DISCUSSION

### Elevated levels of cobamide synthase negatively affect the proton motive force, cell membrane permeability, and cell viability.

Previous attempts to overproduce CobS in E. coli impaired cell growth and triggered the overproduction of the phage shock protein A (PspA) (20), a protein known to be produced under conditions that dissipate the PMF (25–27). These intriguing results suggested that a physiologic excess of cobamide synthase might compromise cell membrane functionality. Among the many functions of the cell membrane is to house electron transport systems that separate protons from electrons, generating a chemical gradient of protons (also known as the proton motive force [PMF]) that fuels cell motility, nutrient transport, ATP synthesis, etc. We investigated the possibility that excess cobamide synthase could dissipate the PMF and negatively affect membrane permeability. To maintain the same experimental framework, the experiments described below were performed with E. coli expressing Salmonella *cobS* alleles encoding proteins that have been previously functionally characterized (24). The 83% identity and 93% similarity between E. coli and S. enterica *cobS* genes (see Fig. S2 in the supplemental material) gave us confidence that the results obtained from experiments described below were meaningful.

10.1128/mBio.02697-21.2 FIG S2 Alignment of the primary sequence of the cobamide synthase (CobS) enzymes encoded by E. coli K-12 MG1655 and S. enterica subsp. *enterica* serovar Typhimurium LT2. The percentages of sequence identity and similarity between these proteins are very high (83% identical, 93% similar). In this alignment, we focus our attention on what is different between these two proteins. Download FIG S2, PDF file, 1.9 MB. Copyright © 2022 Jeter and Escalante-Semerena. 2022 Jeter and Escalante-Semerena. https://creativecommons.org/licenses/by/4.0/ This content is distributed under the terms of the Creative Commons Attribution 4.0 International license.

### (i) Assessment of ethidium bromide (EtBr) efflux.

**FIG 3:**
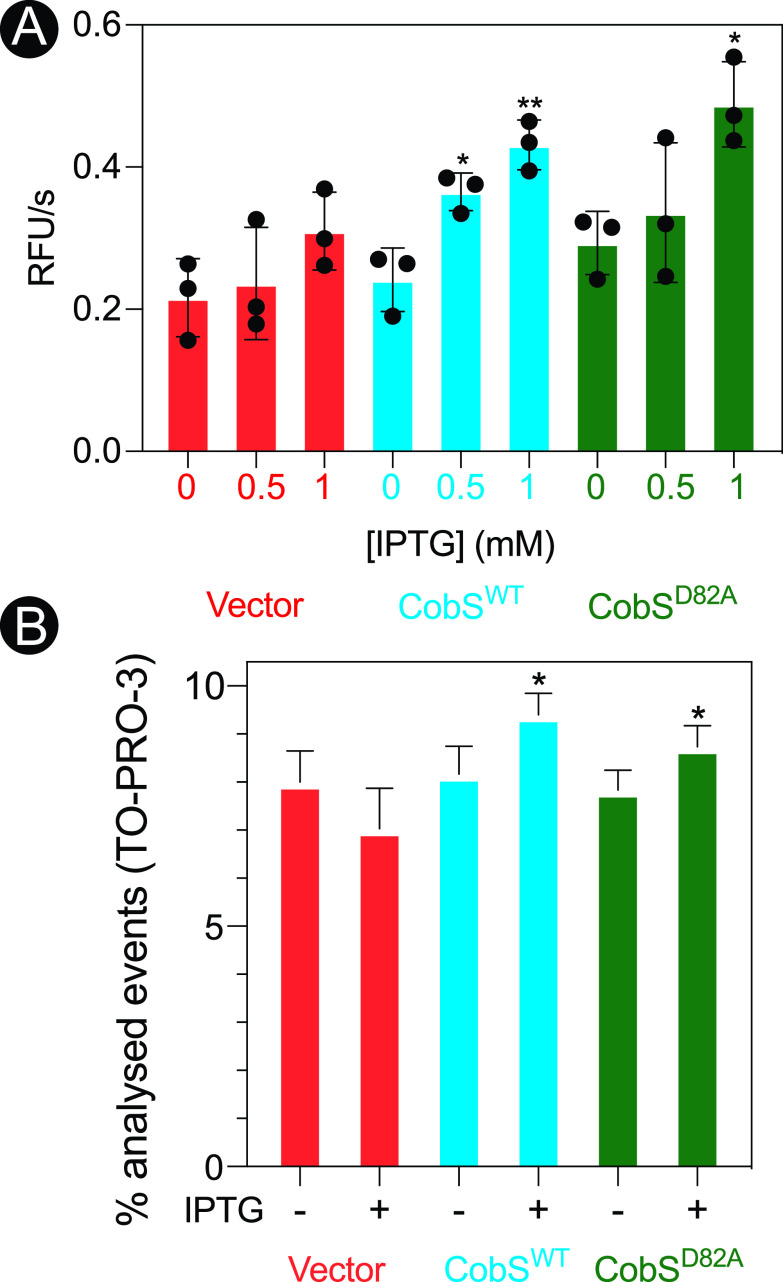
CobS expression compromises membrane stability. (A) E. coli C41(λDE3) cultures synthesizing CobS were grown in a microtiter dish to an OD_600_ of ∼0.4, induced with ITPG (0.5 or 1 mM), and incubated for 30 min at 37°C with shaking. Cells were stained with ethidium bromide, and fluorescence at an excitation of 530 nm and emission of 600 nm was monitored over 3 min. The rate of uptake is expressed as RFU/s as a function of increasing concentrations of IPTG for each condition. Experiments were conducted in biological triplicates, with each experiment containing technical triplicates. Error bars represent the standard error of the mean of technical triplicates. An unpaired Student's *t* test was performed compared to the vector control to determine statistical significance (*, *P* < 0.05; **, *P* = 0.0044). (B) E. coli C41(λDE3) cultures expressing *cobS* were grown to an OD_600_ of ∼0.4, gene expression was induced with ITPG (1 mM), and the cells were incubated for 30 min at 37°C with shaking at 180 rpm. Cells were stained with TO-PRO-3 and analyzed by flow cytometry to determine the number of cells that were permeabilized. Permeabilized cells are shown as percent averages plus the standard error of the mean for each condition tested. A paired *t* test was performed to determine statistical significance compared to the empty vector (*, *P* < 0.05). Cells carrying the empty cloning vector or the *cobS* allele that encoded variant CobS^D82A^ were analyzed as controls.

The first approach we took to determine whether high levels of cobamide synthase affected the PMF was based on the fact that ethidium bromide (EtBr), a fluorescent dye routinely used to stain DNA, is efficiently kept outside the cell by a PMF-driven efflux pump (28). It has been shown that dissipation of the PMF in E. coli leads to the accumulation of EtBr in the cytoplasm, with concomitant intercalation of EtBr into DNA and increased cell fluorescence (29). We used this approach to probe the effect of cobamide synthase overproduction on the PMF. For this purpose, we measured EtBr accumulation in E. coli cells that synthesized wild-type CobS (CobS^WT^ [active]) or D82A mutant (CobS^D82A^ [inactive]) proteins (24). Shown in Fig. 3A are the rates of EtBr accumulation in cells that expressed *cobS* alleles encoding CobS^WT^ or CobS^D82A^ proteins. We measured a significant increase (for CobS^WT^, *P* = 0.015 [*] and *P* = 0.0044 [**]; for CobS^D82A^, *P* = 0.011 [*]) in the rate of EtBr accumulation when the genes encoding CobS^WT^ and CobS^D82A^ were induced, a result that was consistent with PMF dissipation. The effect was not observed in cells harboring the empty cloning vector used to express the *cobS* alleles alluded to. The fact that the enzymatically inactive variant CobS^D82A^ also triggered EtBr accumulation suggested that the presence, not the activity, of the CobS protein was necessary and sufficient to dissipate the PMF.

### (ii) Assessment of cell membrane permeability.

We also probed the integrity of the cell membrane by measuring the uptake of the membrane-impermeable dye thiazole red (also known as TO-PRO-3; Biotium), which is a dye that fluoresces strongly when bound to DNA. The positive charge of this molecule is the main reason why this dye does not diffuse across the cell membrane (30). TO-PRO-3 is a good reporter of membrane permeability in the absence of changes in membrane potential (31). Figure 3B shows the percentage of permeabilized cells in populations expressing CobS^WT^ or CobS^D82A^ 30 min after induction of the *cobS* alleles encoding these proteins. We observed a significant (*P* = 0.0319 [*]) increase in permeability as a result of CobS overproduction (Fig. 3B) compared to an empty cloning vector control (Fig. 3B). We observed the same effect with excess inactive CobS (Fig. 3B; *P* = 0.0351 [*]).

**FIG 4:**
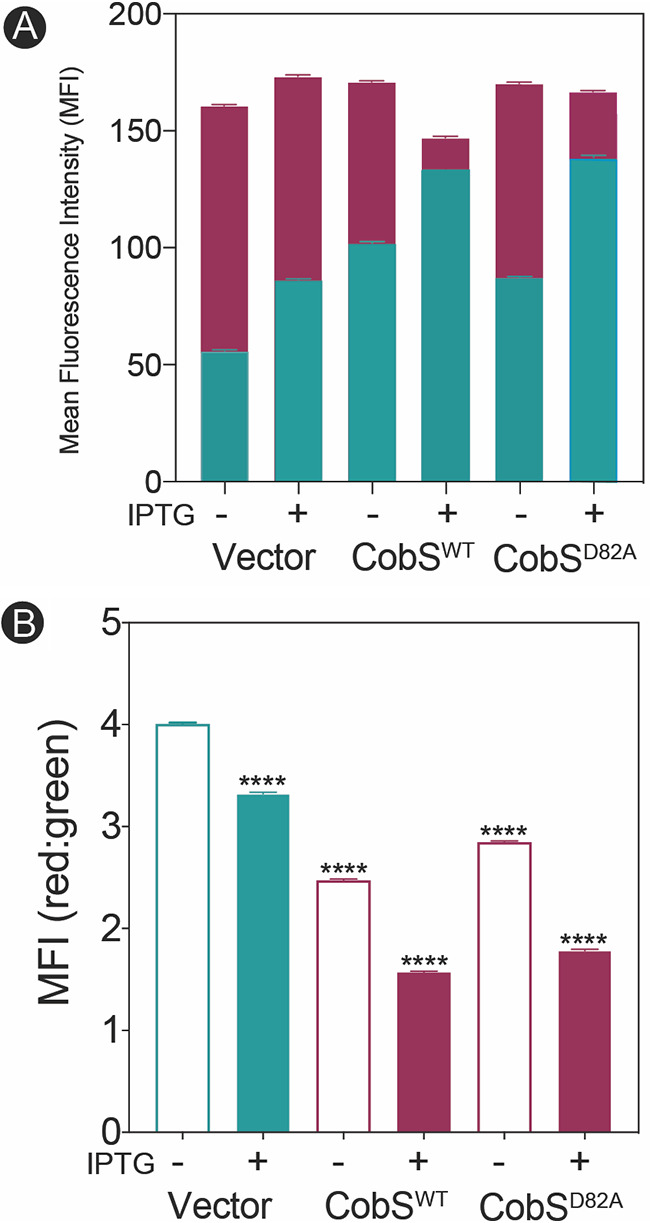
*In vivo* evidence that high CobS levels dissipate the PMF. E. coli C41(λDE3) cultures synthesizing CobS were grown to an OD_600_ of ∼0.4, and gene expression was induced with ITPG (1 mM), followed by a 30-min incubation at 37°C with shaking at 180 rpm. Cells were stained with DiOC_2_(3) and analyzed by flow cytometry to determine membrane depolarization. Cells carrying the empty cloning vector and a vector carrying the *cobS* allele encoding CobS^D82A^ were analyzed as controls. (A) Mean fluorescence intensity (MFI) for both red (filled bars) and green (open bars) fluorescence is shown for each condition. (B) The ratio of red to green fluorescence under each condition is shown. A one-way ANOVA followed by a *post hoc* Bonferroni’s multiple-comparison test was performed to determine the statistical significance between the empty vector control and CobS-synthesizing conditions (****, *P* < 0.0001).

In parallel to monitoring TO-PRO-3 uptake, we assessed the effect of CobS overproduction on membrane potential using the carbocyanine dye 3,3′-diethyloxacarbocyanine iodide [DiOC_2_(3)] (Fig. 4). DiOC_2_(3) initially exhibits green fluorescence, but shifts to red emission as the dye concentrates in the cytoplasm of cells with larger PMF (30). In Fig. 4A, the mean fluorescence intensity (MFI) of red fluorescence (red bars) and green fluorescence (green bars) is displayed for cells expressing CobS^WT^ or inactive variant CobS^D82A^. We observed an increase of green fluorescence in response to CobS^WT^ or CobS^D82A^. In Fig. 4B, the ratio of red to green MFI is shown for the same cell populations analyzed in Fig. 4A. A significant decrease in the ratio of red to green MFI was observed on cells expressing CobS^WT^ and CobS^D82A^ compared to the cells harboring empty cloning vector. In total, the results from panels A and B strongly indicate membrane depolarization.

### (iii) Elevated levels of CobS protein reduce cell viability.

**FIG 5:**
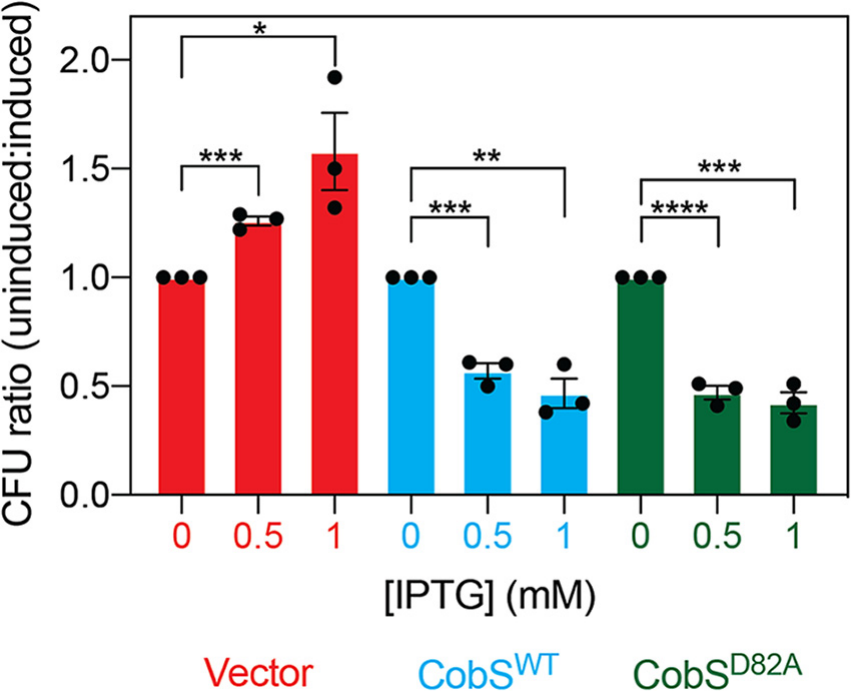
CobS expression decreases cell viability. E. coli C41(λDE3) cultures synthesizing CobS were grown to an OD_600_ of ∼0.4, and gene expression was induced with ITPG (0.5 mM and 1 mM), followed by a 30-min incubation at 37°C with shaking at 180 rpm. Cultures were diluted, and colonies were counted after 16 h of growth at 37°C on LB–1.5% agar plates. Cells carrying the empty cloning vector and a vector carrying the *cobS* allele encoding CobS^D82A^ were included as controls. The ratio of CFU of cultures without induction compared to cultures with induction is shown. An unpaired Student's *t* test was performed to determine statistical significance between induced and noninduced conditions (*, *P* = 0.031; **, *P* = 0.0014; ***, *P* = 0.003; ****, *P* < 0.0001).

In addition to monitoring the effects of elevated levels of CobS on the membrane, we assessed the consequences of membrane dysfunction on cell viability (Fig. 5). We observed a significant decrease in colony-forming units (CFU) as we increased the level of CobS^WT^ (*P* = 0.0003 for 0.5 mM isopropyl-β-d-thiogalactopyranoside [IPTG] and *P* = 0.0014 for 1 mM IPTG) or CobS^D82A^ (*P* < 0.0001 for 0.5 mM IPTG, and *P* = 0.0003 for 1 mM IPTG). Conversely, we saw an increase of CFU in strains expressing a vector control. These results suggested that cells must control *cobS* expression and probably couple it to the synthesis of other components to avoid compromising cell viability.

### Cells overexpressing *cobS* exhibit atypical morphology.

**FIG 6:**
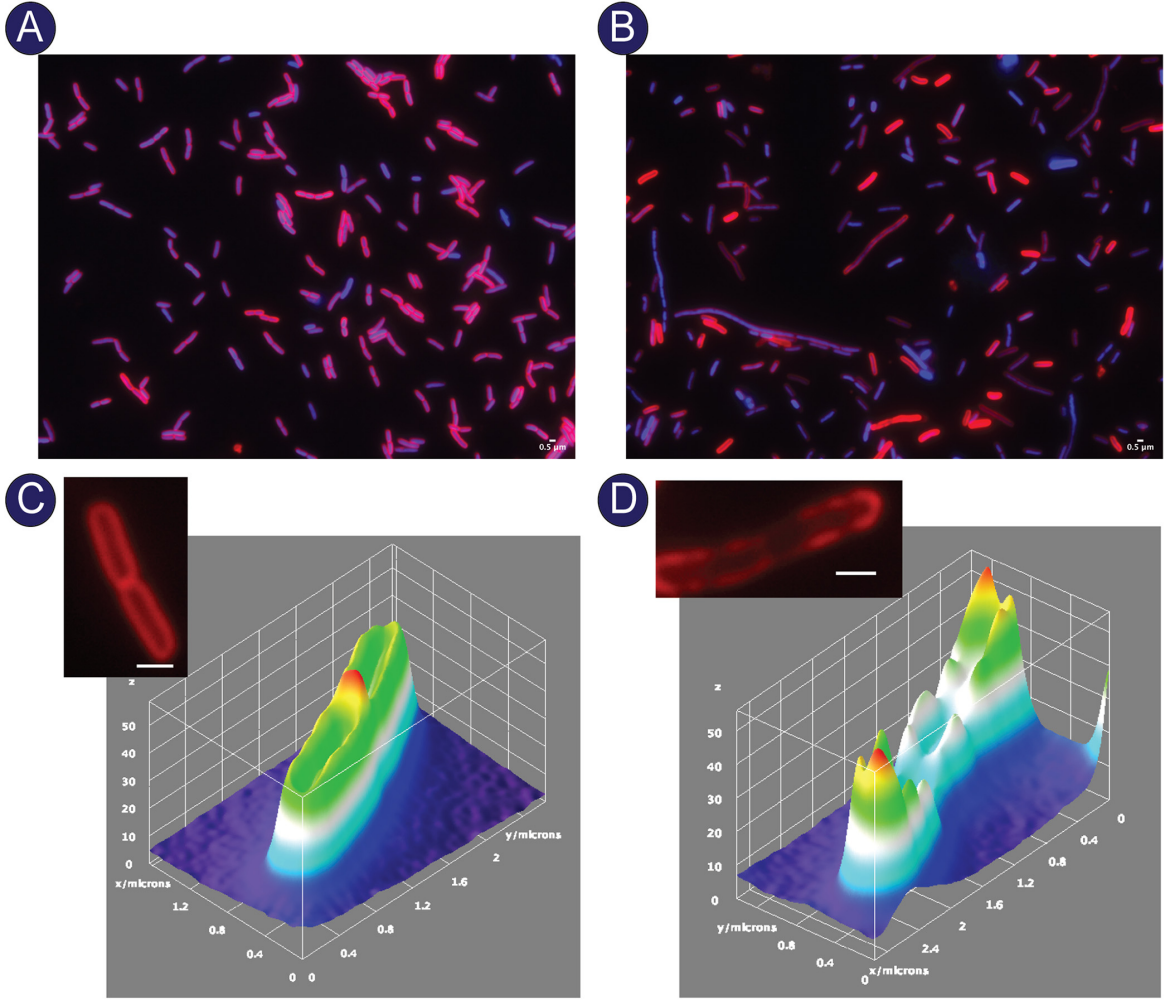
Microscopic evaluation of excess CobS on cell morphology. E. coli C41(λDE3) cultures harboring an empty vector (A) or synthesizing CobS (B) were grown to an OD_600_ of ∼0.4, and gene expression was induced with ITPG (1 mM), followed by a 30-min incubation at 37°C with shaking at 180 rpm. Cells were stained with DAPI and FM4-64. (C) Surface intensity plot of FM6-64 fluorescence from a cell carrying empty vector. The stained cell used to generate the plot is shown. (D) Surface intensity plot of FM6-64 fluorescence from a cell synthesizing CobS. The stained cell used to generate the plot is shown. The scale is 0.5 μm. In panels C and D, the *z* axis displays fluorescence intensity, while the *x* and *y* axes are plotted in micrometers.

To visualize the effects of *cobS* overexpression on the cell membrane, we used fluorescence microscopy with the lipophilic stain FM4-64, which is a membrane stain that exhibits uniform staining in wild-type cells. Membrane perturbations disrupt the uniformity of staining, making FM4-64 an effective indicator of membrane abnormalities (32, 33). When stained with FM4-64, cells harboring an empty vector exhibited uniform staining of the membrane (Fig. 6A and C; for additional images, see Fig. S5 in the supplemental material). As expected, cells synthesizing CobS exhibited nonuniform FM4-64 staining of the membrane (Fig. 6B and D; for additional images see Fig. S6 in the supplemental material). The significant membrane perturbations caused by elevated levels of CobS were visualized by three-dimensional fluorescence intensity spectrum (Fig. 6D). Compared to cells harboring empty vector (Fig. 6C), CobS-synthesizing cells exhibited a punctate staining around the membrane (Fig. 6D). This phenomenon was further exemplified by the ranges of fluorescent intensity across the cell perimeter depicted by the spectrum. In contrast, the three-dimensional spectrum of cells harboring empty vector shows uniform intensities around the perimeter of the cell, with a slight but clear increase at the divisional septum (Fig. 6C).

10.1128/mBio.02697-21.5 FIG S5 Wild-type cells exhibit uniform membrane staining. E. coli C41(λDE3) cultures harboring empty vector were grown to an OD_600_ of ∼0.4 and induced with ITPG (1 mM), followed by a 30-min incubation at 37°C with shaking at 180 rpm. Cells were stained with DAPI and FM4-64. Surface intensity plots of FM6-64 fluorescence from cells harboring empty vector are shown. Stained cells used to generate plots are shown. Scale bars, 0.5 μm. On surface intensity plots, the *z* axis displays fluorescence intensity, while the *x* and *y* axes are plotted in micrometers. Panels A, B, C, and D each show an individual cell and the corresponding FM4-64 fluorescence surface intensity plot. Download FIG S5, PDF file, 2.9 MB. Copyright © 2022 Jeter and Escalante-Semerena. 2022 Jeter and Escalante-Semerena. https://creativecommons.org/licenses/by/4.0/ This content is distributed under the terms of the Creative Commons Attribution 4.0 International license.

10.1128/mBio.02697-21.6 FIG S6 Cells synthesizing CobS display membrane perturbations. E. coli C41(λDE3) cultures synthesizing CobS were grown to an OD_600_ of ∼0.4, and gene expression was induced with ITPG (1 mM), followed by a 30-min incubation at 37°C with shaking at 180 rpm. Cells were stained with DAPI and FM4-64. Surface intensity plots of FM6-64 fluorescence from cells synthesizing CobS are shown. Stained cells used to generate plots are shown. Scale bars, 0.5 μm. On surface intensity plots, the *z* axis displays fluorescence intensity, while the *x* and *y* axes are plotted in micrometers. Panels A, B, C, and D each show an individual cell and the corresponding FM4-64 fluorescence surface intensity plot. Download FIG S6, PDF file, 3.4 MB. Copyright © 2022 Jeter and Escalante-Semerena. 2022 Jeter and Escalante-Semerena. https://creativecommons.org/licenses/by/4.0/ This content is distributed under the terms of the Creative Commons Attribution 4.0 International license.

A surprising finding exhibited by CobS-synthesizing cells can be seen in Fig. 6B. Populations of these cells mostly lacked divisional septa, and numerous elongated cells were seen, indicating potential effects of high CobS activity levels on cell division and DNA replication. Cases of cell filamentation due to perturbations of the assembly of the divisome and DNA replication have been extensively studied (34–37). Intact divisional septa were clearly visualized throughout the population in cells harboring empty vector (Fig. 6A). Notably, the PMF is known to play an important role in proper localization of the divisome (38, 39). The work reported here indicates significant membrane depolarization as a result of elevated levels of CobS; thus, we posit that defective cell division may be ultimately a consequence of CobS overproduction in asynchrony with other Cob proteins.

### Elevated levels of the AdoCbl-P phosphatase (CobC) or phage shock protein (PspA) relieve membrane stress caused by elevated CobS levels.

We sought to alleviate the membrane stress observed as a consequence of elevated levels of CobS by coexpressing *pspA* or *cobC*. As mentioned above, previous work from our group showed that an increased level of PspA was observed in response to CobS overproduction (20). Since PspA has been reported to play a role in PMF maintenance (reviewed in reference 40), we investigated whether the negative effect of CobS overproduction could be minimized by increasing the level of PspA. As for CobC, its role in Cba biosynthesis is firmly established as the enzyme that catalyzes the final step of the pathway by cleaving the phosphate from AdoCba-P, the product of the CobS reaction (Fig. 2) (23). The CobC proteins from E. coli and Vibrio parahaemolyticus have been crystallized as dimers (PDB accession no. 6E4B and 3HJG, respectively). We performed hydropathy analysis of the E. coli protein using the TMPred software program. The results of this analysis identified a single transmembrane domain between residues 143 and 168 (see Fig. S7 in the supplemental material), making it a potential candidate for interactions with CobS.

10.1128/mBio.02697-21.7 FIG S7 S. enterica CobC is homologous to Escherichia coli CobC. (A) Alignment of S. enterica and E. coli CobC protein sequences. Identical and similar amino acids are highlighted in red and yellow, respectively. The secondary structure of E. coli CobC is shown above the sequences. Alignments were generated using ESPript (51). (B) The crystal structure of E. coli CobC (PDB accession no. 6E4B). The predicted transmembrane domain is shown in purple. The N and C termini are shown in green and red, respectively. This structure was illustrated using UCSF Chimera (52). The transmembrane domain was predicted by a hydropathy analysis done using the online ExPASy TMpred software program (https://embnet.vital-it.ch/software/TMPRED_form.html). Download FIG S7, PDF file, 3.0 MB. Copyright © 2022 Jeter and Escalante-Semerena. 2022 Jeter and Escalante-Semerena. https://creativecommons.org/licenses/by/4.0/ This content is distributed under the terms of the Creative Commons Attribution 4.0 International license.

**FIG 7:**
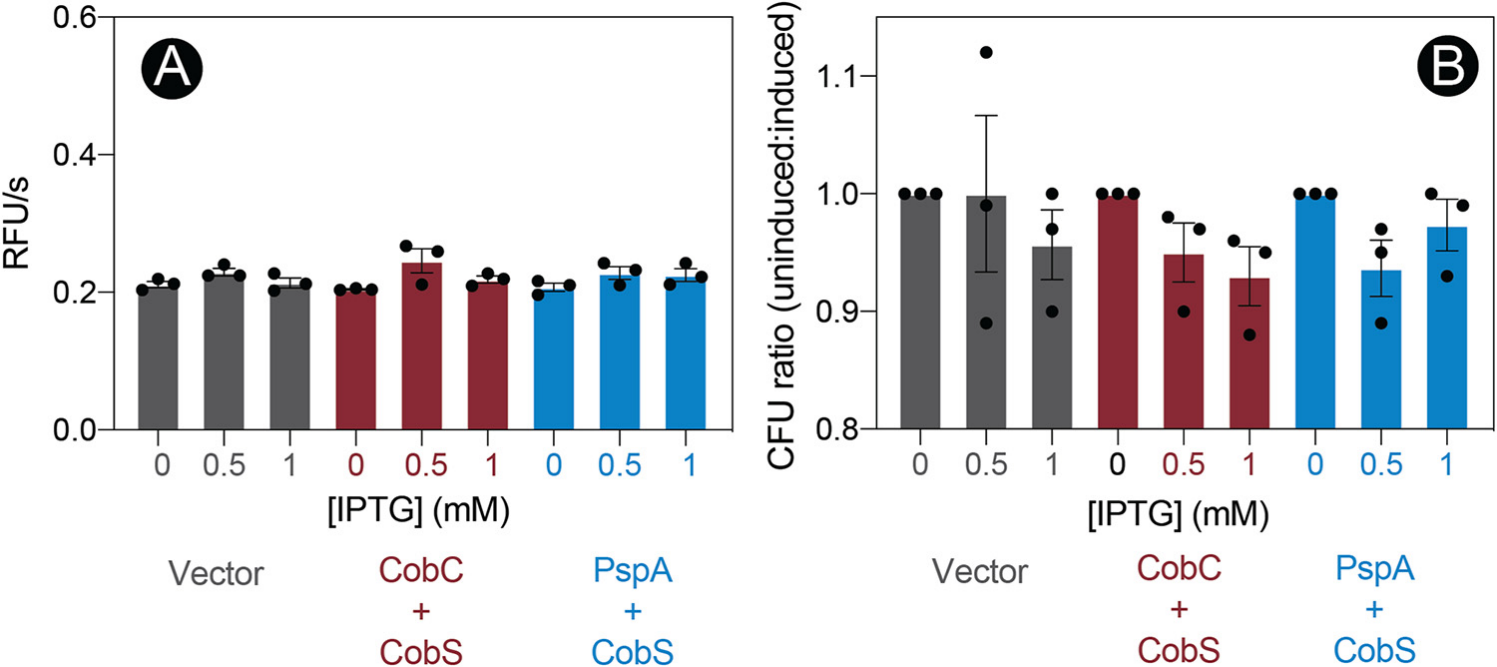
Coexpression of CobC or PspA ameliorates CobS-induced membrane instability. (A) E. coli C41(λDE3) cultures synthesizing CobC and CobS or PspA and CobS were grown in a microtiter dish to an OD_600_ of ∼0.4, induced with ITPG (0.5 or 1 mM), and incubated for 30 min at 37°C with shaking. Cells were stained with ethidium bromide, and fluorescence at an excitation of 530 nm and emission of 600 nm was monitored over 3 min. The rate of uptake is expressed as RFU/s as a function of increasing concentrations of IPTG for each condition. Experiments were conducted in biological triplicates, with each experiment containing technical triplicates. Error bars represent the standard error of the mean of technical triplicates. No significant differences were observed across IPTG concentrations, as determined by an unpaired Student's *t* test. (B) E. coli C41(λDE3) cultures synthesizing CobS and CobC or PspA and CobS were grown as previously described. Cultures were diluted, and colonies were counted after 16 h of growth at 37°C on LB–1.5% agar plates. Cells carrying the empty cloning vector were included as a control. The ratio of CFU of cultures without induction compared to cultures with induction is shown. An unpaired Student's *t* test determined there was no significant difference between strains and across IPTG concentrations.

To determine whether increased levels of CobC or PspA could counteract the negative effects of CobS overproduction, we used vector pRSFDUET-1, which allowed us to coexpress CobC and CobS or PspA and CobS. Using this expression system, we repeated the EtBr assay. Shown in Fig. 7A are the rates of EtBr accumulation by cells harboring the empty vector versus the rate of accumulation by cells synthesizing CobC plus CobS or PspA plus CobS across a range of inducer (IPTG) concentrations. The results clearly indicated that the balance of CobC or PspA expression with CobS ameliorated the detrimental effects we observed with CobS alone (Fig. 3). As expected, balanced coexpression of CobC or PspA with CobS also improved cell viability (Fig. 7B). These results were in stark contrast to those shown in Fig. 5, where we observed a drastic decrease in cell viability as a function of *cobS* induction. These results suggested that CobC or PspA somehow blocked the negative effects of an elevated CobS level.

**FIG 8:**
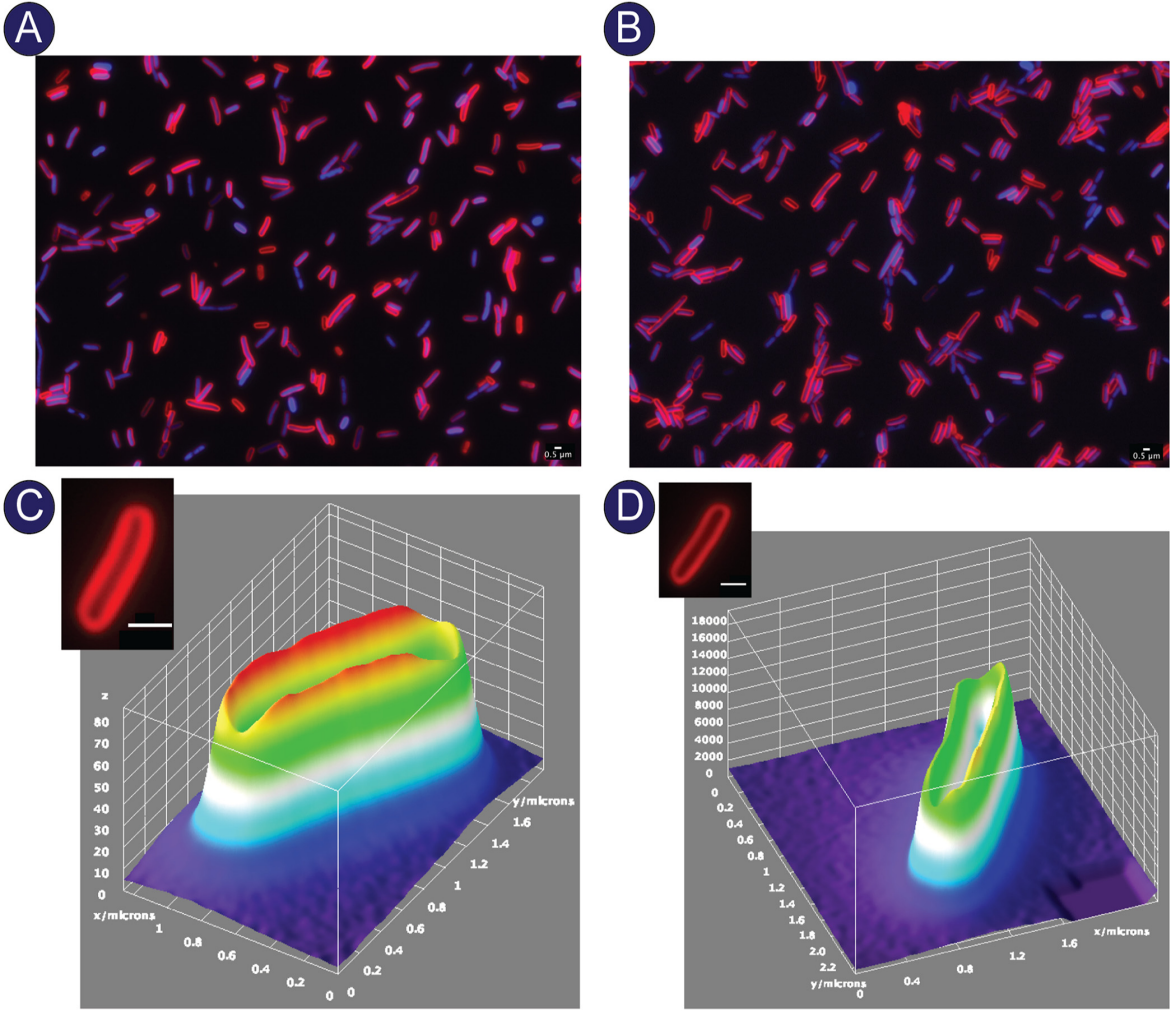
Microscopic evaluation of coexpression of CobC or PspA and CobS on cell morphology. E. coli C41(λDE3) cells synthesizing CobC and CobS (A) or synthesizing PspA and CobS (B) were grown to an OD_600_ of ∼0.4, and gene expression was induced with ITPG (1 mM), followed by a 30-min incubation at 37°C with shaking at 180 rpm. Cells were stained with DAPI and FM4-64. (C) Surface intensity plot of FM6-64 fluorescence from a cell synthesizing CobC and CobS. The stained cell used to generate the plot is shown. (D) Surface intensity plot of FM6-64 fluorescence from a cell synthesizing PspA and CobS. The stained cell used to generate the plot is shown. Scale bars, 0.5 μm. In panels C and D, the *z* axis displays fluorescence intensity, while the *x* and *y* axes are plotted in micrometers.

To visualize the effects of coexpression of *cobC* or *pspA* with *cobS*, we utilized fluorescence microscopy. Cells synthesizing CobC (Fig. 8C; for additional images, see Fig. S8 in the supplemental material) or PspA (Fig. 8D; for additional images, see Fig. S9 in the supplemental material) in addition to CobS exhibited uniform staining of the membrane, as shown by the three-dimensional fluorescence intensity spectrum. Populations of cells synthesizing CobC and CobS (Fig. 8A) or PspA and CobS (Fig. 8B) showed more intact septa and fewer elongated cells compared to cells synthesizing only CobS (Fig. 6B). These results were consistent with improved cell viability and membrane integrity exhibited when CobS was coexpressed with CobC or PspA.

10.1128/mBio.02697-21.8 FIG S8 Cells coexpressing *cobC*^+^ and *cobS*^+^ exhibit uniform membrane staining. E. coli C41(λDE3) cultures synthesizing CobC^WT^ and CobS^WT^ were grown to an OD_600_ of ∼0.4, and induced with ITPG (1 mM), followed by a 30-min incubation at 37°C with shaking at 180 rpm. Cells were stained with DAPI and FM4-64. Surface intensity plots of FM6-64 fluorescence from cells harboring empty vector are shown. Stained cells used to generate plots are shown. Scale bars, 0.5 μm. On surface intensity plots, the *z* axis displays fluorescence intensity, while the *x* and *y* axes are plotted in micrometers. Panels A, B, C, and D each show an individual cell and the corresponding FM4-64 fluorescence surface intensity plot. Download FIG S8, PDF file, 4.7 MB. Copyright © 2022 Jeter and Escalante-Semerena. 2022 Jeter and Escalante-Semerena. https://creativecommons.org/licenses/by/4.0/ This content is distributed under the terms of the Creative Commons Attribution 4.0 International license.

10.1128/mBio.02697-21.9 FIG S9 Cells coexpressing *pspA*^+^ and *cobS*^+^ exhibit uniform membrane staining. E. coli C41(λDE3) cultures synthesizing PspA^WT^ and CobS^WT^ were grown to an OD_600_ of ∼0.4 and induced with ITPG (1 mM), followed by a 30-min incubation at 37°C with shaking at 180 rpm. Cells were stained with DAPI and FM4-64. Surface intensity plots of FM6-64 fluorescence from cells harboring empty vector are shown. Stained cells used to generate plots are shown. Scale bars, 0.5 μm. On surface intensity plots, the *z* axis displays fluorescence intensity while the *x* and *y* axes are plotted in micrometers. Panels A, B, C, and D each show an individual cell and the corresponding FM4-64 fluorescence surface intensity plot. Download FIG S9, PDF file, 4.2 MB. Copyright © 2022 Jeter and Escalante-Semerena. 2022 Jeter and Escalante-Semerena. https://creativecommons.org/licenses/by/4.0/ This content is distributed under the terms of the Creative Commons Attribution 4.0 International license.

### CobS-dependent association of CobC with liposomes.

**FIG 9:**
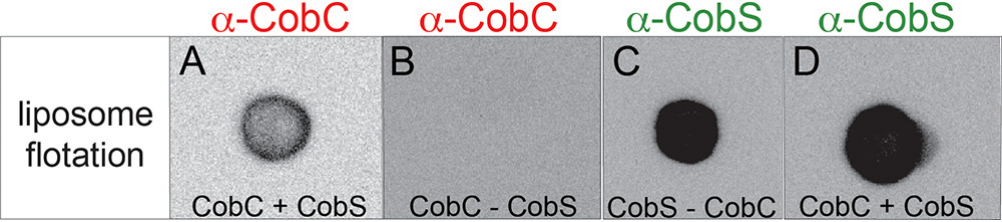
CobC interacts with CobS embedded in phospholipid bilayer. Shown is dot blot analysis of CobC-CobS proteoliposome complex. (A) CobC was incubated with CobS-containing proteoliposomes prior to flotation on a Histodenz gradient. (B) CobC was incubated with empty proteoliposomes. Polyclonal rabbit antibodies against CobC were used to probe for CobC in experiments described in panels A and B. Panels C and D show dot blots of CobS proteoliposomes probed with polyclonal rabbit antibodies against CobS in the absence (C) or presence (D) of CobC.

Given the restoration of cell viability and membrane stability observed when *cobC* was coexpressed with *cobS*, we sought to determine whether CobS could affect the localization of CobC to a lipid bilayer. To investigate this possibility, we employed a liposome flotation strategy. Purified CobC was incubated with liposomes that were or were not preloaded with CobS. The resulting liposome suspensions were subjected to ultracentrifugation through a Histodenz gradient. We hypothesized that if CobC interacted with CobS proteoliposomes, it would move through the density gradient and “float” to the top layer. The presence of CobC or CobS was detected using a dot blot Western blot assay that employed polyclonal rabbit antibodies against each protein (i.e., anti-CobC or anti-CobS). When probed using anti-CobC antibodies, we observed that CobC associated with CobS-containing proteoliposomes (Fig. 9A), but not with empty liposomes (Fig. 9B). The presence of CobS in the proteoliposome did not depend on the presence of CobC (Fig. 9C and D) (24).

### Concluding remarks.

Here, we provide insights into how important it is for Escherichia coli, and probably many other cobamide-producing prokaryotes, to avoid uncontrolled increases of cobamide synthase (CobS) enzyme that is embedded in the cell membrane. Based on the data reported herein, we conclude that if CobS levels are not kept under control, the cell faces a collapse of its energy charge, increases in cell permeability, as well as probably the inability to generate a divisome, and ultimately death. We speculate that CobS may form a pore-like structure through which protons may be lost and that the CobC and PspA somehow prevent proton leakage.

We propose that CobS may be one of several integral cell membrane proteins of the cobamide biosynthetic pathway whose role is to anchor a large multienzyme complex. We posit that, at a minimum, such a complex includes the polytopic AdoCbi-P synthase (CbiB) enzyme that catalyzes the first step of the nucleotide loop assembly (NLA) pathway (11), the CobC phosphatase that catalyzes the last step of the NLA pathway (23), and the cobamide synthase (CobS). Much is unknown about the assembly of such a putative multienzyme complex, but we at least know that CobS is needed to recruit CobC. A complex that assembles the nucleotide loop from the precursors cobinamide and DMB would involve CobS, CobU, and CobC. The polytopic CbiB (AdoCbi-P synthase) enzyme links *de novo* corrin ring biosynthesis to the NLA pathway complex—hence, it would likely be part of the proposed multienzyme complex.

It should not go unnoticed that in all sequenced genomes of cobamide producers, CbiB and CobS are polytopic proteins, a fact that points at an unknown, strong positive selection that has maintained cobamide synthesis in integral association with the cell membrane (whether bacterial or archaeal in nature) throughout evolution. To date, not one CobS or CbiB homologue found in genome databases has a sequence that would imply a cytosolic location. Clearly, cobamide biosynthesis poses chemical and physiological challenges to the cell, which apparently can only be met by involving the cell membrane.

## MATERIALS AND METHODS

### Bacterial strains, culture media, and chemicals.

Bacterial strains used in this study are listed in Table 1. Escherichia coli C41(λDE3) (41) strains were grown at 37°C on lysogeny broth (LB; Difco) (42, 43). Escherichia coli C41(λDE3) was used for protein overexpression, membrane assessments, and cell viability determinations. E. coli K-12 strain DH5α (New England Biolabs) was used for plasmid construction. Antibiotics for all media were used at the following concentrations: ampicillin, 100 μg mL^−1^; kanamycin, 50 μg mL^−1^. All chemicals were purchased from Sigma-Aldrich unless otherwise noted, such as isopropyl-β-d-1-thiogalactopyranoside (IPTG; Gold BioTechnology), glycerol (Fisher), 4-(2-hydroxyethyl)-1-piperazineethanesulfonic acid buffer (HEPES; Gold BioTechnology), 3-[(3-cholamidopropyl)dimethylammonio]-1-propanesulfonate detergent (CHAPS, Gold BioTechnology), 1-palmitoyl-2-oleoyl-glycero-3-phosphocholine (POPC; Avanti Polar Lipids), 1-palmitoyl-2-oleoyl-*sn-*glycero-3-phosphoethanolamine (POPE; Avanti Polar Lipids), 1-palmitoyl-2-oleoyl-*sn-*glycero-3-phoshpo-l-serine (POPS; Avanti Polar Lipids), Lissamine rhodamine B 1,2-dihexadecanoyl-*sn-*glycero-3-phosphoethanolamine (Rh-DHPE; Molecular Probes) Oriole fluorescent gel stain (Bio-Rad Laboratories). The chemical structures of the lipids and fluorophore used in this study are shown in Fig. S3 in the supplemental material.

**TABLE 1:** Strains and plasmids used in this work

Strain or plasmid	Genotype	Reference or source
Strain		
E. coli JE6663 C41(λDE3)	F^−^ *ompT hsdSB*(r_B_^−^ m_B_^−^) *gal dcm* (λDE3)	Laboratory collection
Plasmids		
pTEV5	Overexpression vector that fuses N terminus of protein of interest to H_6_ tag, which can be removed by rTEV protease; *bla*^+^	46
pTEV16	Overexpression vector that fuses N terminus of protein of interest to His_6_ tag, which can be removed by rTEV protease; *bla*^+^	45
pRSFDUET-1	Overexpression vector that allows for coexpression of 2 ORFs; *kan*^+^	Novagen
pCOBS5	*S.* Typhimurium *cobS^+^* cloned into vector pET-15b; plasmid encodes CobS protein with noncleavable His tag fused to its N terminus	21
pCOBS120	*S.* Typhimurium *cobS*^+^ cloned into vector pTEV16	
pCOBS121	*S.* Typhimurium *cobS1452*; this allele encodes variant CobS^D82A^, and allele was cloned into vector pTEV16	
pCOBS122	*S.* Typhimurium *cobS*^+^ MCS2/pRSFDUET-1	
pCOBS123	*S.* Typhimurium *cobC*^+^ MCS1; *S.* Typhimurium *cobS*^+^ MCS2/pRSFDUET-1	
pCOBS124	E. coli *pspA*^+^ MCS1 *S.* Typhimurium *cobS*^+^ MCS2/pRSFDUET-1	
pCOBC106	*S.* Typhimurium *cobC*^+^ cloned into vector pTEV5	

10.1128/mBio.02697-21.3 FIG S3 Chemical structures of the lipids and fluorophore used in this study. The lipids and the percentages used to generate liposomes are indicated. POPC (1-palmitoyl-2-oleoyl-glycero-3-phosphocholine), POPS (1-palmitoyl-2-oleoyl-*sn*-glycero-3-phospho-l-serine), POPE (1-palmitoyl-2-oleoyl-*sn*-glycero-3 phosphoethanolamine), and Rh-DHPE (Lissamine rhodamine B 1,2-dihexadecanoyl-*sn*-glycero-3-phosphoethanolamine) were used to generate liposomes. Download FIG S3, PDF file, 1.0 MB. Copyright © 2022 Jeter and Escalante-Semerena. 2022 Jeter and Escalante-Semerena. https://creativecommons.org/licenses/by/4.0/ This content is distributed under the terms of the Creative Commons Attribution 4.0 International license.

### Plasmid construction.

Plasmids used in this study are listed in Table 1. Primers were synthesized by Integrated DNA Technologies, Inc. (IDT [Coralville, IA]), and are listed in Table 2. Genes were amplified from S. enterica genomic DNA using Phusion DNA polymerase (Thermo Fisher) per the manufacturer’s instructions. Restriction enzymes were purchased from Fermentas. The BspQI restriction enzyme was purchased from New England BioLabs.

**TABLE 2:** Primers used in this work^*a*^

Primer name	Primer sequence (5′→3′)
cobS pCV1 F	NNGCTCTTCNTTCATGAGTAAGCTGTTTTGGGC
cobS pCV1 R	NNGCTCTTCNTTATAACAGAGCCAGCAGAA
CobS NdeI F	NNNNCATATGATGAGTAAGCTGTTTTGG
CobS KpnI R	NNNNGGTACCTCATAACAGAGCCAGCAG
CobC EcoRI F	NNNGAATTCGAGGAATACCATGCGA
CobC HindIII R	NNNAAGCTTTCACTCAGGCCGCCA
CobC NheI F	NNNGCTAGCGAGGAATACCATGCGA
CobC SacI R	NNNGAGCTCTCACTCAGGCCGCCA
PspA EcoRI	NNNNGAATTCATGGGTATTTTTTCTCGC
PspA HindIII	NNNNAAGCTTTTATTGATTGTCTTGCTT

a All primers used in this work were synthesized by Integrated DNA Technologies (IDT, Coralville, IA).

### Plasmids pCOBS120 and pCOBS121.

Plasmids pCOBS120 and pCOBS121 were used for expression studies in E. coli C41(λDE3). Plasmid pCOBS120 encoded CobS^WT^, and plasmid pCOBS121 encoded variant CobS^D82A^. Both plasmids provided resistance to ampicillin and were constructed by the BspQI high-efficiency cloning method described elsewhere (44, 45), using primers cobS pCV1 F and cobS pCV1 R to amplify *cobS^+^* and primers cobS pCV1 F and cobS pCV1 R to amplify the *cobS* allele encoding CobS^D82A^. *cobS* alleles were inserted between the pair of BspQI sites of pTEV16 (45).

### Plasmids pCOBS122, pCOBS123, and pCOBS124.

Plasmids pCOBS122, pCOBS123, and pCOBS124 were constructed using traditional cloning methods with restriction enzyme digestion. The *cobS^+^* allele was amplified from S. enterica genomic DNA using primers CobS NdeI F and CobS KpnI R. Amplified *cobS^+^* and pRSFDUET-1 were digested with NdeI and KpnI as per the manufacturer’s directions. T4 DNA ligase was used according to the manufacturer’s instructions to insert *cobS^+^* into MCS2 of pRSFDUET-1, yielding pCOBS122. pCOBS122 is 4,589 bp long and encodes resistance to kanamycin. Transcription of *cobS^+^* was induced by the addition of IPTG, which triggered the synthesis of T7 polymerase. CobS overproduced from pCOBS122 contained a C-terminal S tag. The *cobC*^+^ allele was amplified from S. enterica using primers CobC EcoRI F and CobC HindIII R. Amplified *cobC^+^* and pCOBS122 were digested with EcoRI and HindIII according to the manufacturer’s instructions and ligated together using T4 DNA ligase, as mentioned above, yielding pCOBS123. pCOBS123 is 5,208 bp long and encodes kanamycin resistance. *cobC^+^* transcription was induced by the addition of IPTG, which triggered the synthesis of T7 polymerase. CobC produced using plasmid pCOBS123 contained an N-terminal 6-histidine tag (H_6_-CobC). The *pspA^+^* allele was amplified from E. coli K-12 strain MG1655 using primers PspA EcoRI F and PspA HindIII R. Amplified *pspA^+^* and pCOBS122 were digested with EcoRI and HindIII according to the manufacturer’s instructions and ligated together using T4 DNA ligase, yielding pCOBS124. Plasmid pCOBS124 is 5,258 bp long and encodes kanamycin resistance. Expression of *pspA^+^* was induced by the addition of IPTG, which triggered the synthesis of T7 polymerase. PspA produced using plasmid pCOBS123 contained an N-terminal 6-hisitidine tag.

### Plasmid pCOBC106 and pTEV5 NheI and SacI.

Plasmid pCOBC106 was constructed using traditional cloning methods with restriction enzyme digestion. The *cobC^+^* allele was amplified from S. enterica genomic DNA using primers CobC NheI F and CobC SacI R. Amplified *cobC^+^* and vector pTEV5 (46) were digested according to the manufacturer’s directions using restriction enzymes NheI and SacI. The *cobC^+^* allele was ligated into pTEV5 using T4 DNA ligase as per the manufacturer’s instructions. Plasmid pCOBC106 was used for protein overproduction. Plasmid pCOBC106 is 5,953 bp long and encodes resistance to ampicillin. *cobC^+^* expression was induced by the addition of IPTG, which triggered the synthesis of T7 polymerase. CobC synthesized from pCOBC106 yielded CobC with a hexahistidine tag fused to its N terminus (H_6_-CobC).

### CobS protein overproduction and purification.

Detailed protocols for the overproduction and purification of CobS can be found in reference 24.

### CobC protein overproduction and purification.

Wild-type CobC protein was overproduced from plasmid pCOBC106 in strain JE6663 [*E. coli* C41(λDE3)] in 1-L cultures of Terrific Broth (47). Protein synthesis was induced by the addition of IPTG at a final concentration of 1 mM in mid-log-phase cultures (optical density at 600 nm [OD_600_] of ∼0.6) growing at 37°C with shaking at 180 rpm in an Innova44 (New Brunswick Scientific) gyratory incubator. After induction, cultures were grown for 17 h at 24°C with shaking at 180 rpm. Cultures were harvested by centrifugation at 4°C for 15 min at 6,000 × *g* in an Avanti J20-XPI refrigerated centrifuge equipped with a JLA-8.1000 rotor. Pelleted cells were stored at −20°C until used. Frozen cells were thawed on ice and resuspended in HEPES buffer (50 mM, pH 7.5) containing NaCl (0.5 M) and imidazole (20 mM) at a ratio of 20% cell weight to buffer volume. Lysozyme (1 μg/mL) and DNase I (25 μg/mL) were added to the cell suspension, and the mixture was incubated on ice for 10 min. Cells were lysed by sonication, and phenylmethylsulfonyl fluoride (PMSF) was added to the cell lysate at a final concentration of 0.5 mM. Cellular debris was removed by centrifugation at 4°C for 30 min at 40,000 × *g* in an Avanti J-251 centrifuge (Beckman Coulter) equipped with a JA 25.25 rotor. Clarified extract was filtered using a 0.45-μm-pore syringe filter unit and applied to a 2-mL HisPur nickel-nitrilotriacetic acid (Ni-NTA) affinity column (Thermo Fisher Scientific). The column was washed with 10 column volumes of HEPES buffer (25 mM, pH 7.5) containing NaCl (0.5 M) and imidazole (20 mM) and 6 column volumes of HEPES buffer (25 mM, pH 7.5) containing NaCl (0.5 M) and imidazole (40 mM). H_6_-CobC was eluted with 6 column volumes of HEPES buffer (25 mM, pH 7.5) containing NaCl (0.5 M) and imidazole (0.5 M). Fractions were collected throughout the wash and elution steps, and H_6_-CobC purification was monitored by SDS-PAGE compared to Precision Plus protein standards (Bio-Rad). Fractions containing H_6_-CobC were pooled and dialyzed against HEPES buffer (25 mM, pH 7.5) containing NaCl (0.5 M) to remove imidazole. H_6_-CobC was then dialyzed against HEPES buffer (25 mM, pH 7.5) in three additional steps with decreasing concentrations of NaCl down to 0.15 M. Purified H_6_-CobC was flash frozen in liquid nitrogen and stored at –80°C until used. The protein concentration was measured using a Bradford assay kit (Bio-Rad laboratories).

### Liposome preparation and protein reconstitution.

Details of the protocols used for the preparation of liposomes and for the insertion of CobS into liposomes can be found in reference 24. Lipids used for this purpose and the compositions used are shown in Fig. S3.

### Liposome flotation assay.

To probe for interactions between CobC and CobS or CobC and the lipid bilayer, liposome flotation assays using CobS-containing proteoliposomes and empty liposomes were employed. Purified CobC protein (final concentration of 10 μM) was incubated with 100 μL of a 1.4 mM lipid solution of CobS proteoliposomes or empty liposomes for 1 h at 24°C in a RotoBot (Benchmark). This mixture was mixed with an equal volume with HEPES buffer (20 mM, pH 7.4) containing NaCl (0.15 M), Histodenz (80% [wt/vol]), and glycerol (10% [vol/vol]) and deposited in a Beckman Coulter ultracentrifuge tube (polyallomer, 11 by 34 mm). A 2.5-mL overlay of HEPES (20 mM, pH 7.4) containing NaCl (0.15 M), Histodenz (30% [wt/vol]), and glycerol (10% [vol/vol]) was applied, followed by a 150-μL overlay of HEPES buffer (20 mM, pH 7.4) containing NaCl (150 mM), and glycerol (10% [vol/vol]). The gradient was subjected to centrifugation at 4°C for 3 h at 214,000 × *g* in a refrigerated Beckman Coulter Optima MAX-XP ultracentrifuge using a TLS-55 rotor. The top layer was harvested, and the lipid concentration was determined by rhodamine fluorescence. A standard curve was generated by measuring the fluorescence of a series of rhodamine concentrations with a SpectraMax Gemini EM microplate reader (Molecular Devices) at an excitation of 540 nm and emission of 586 nm.

### Dot blot analysis.

To confirm the presence of CobC in liposome flotation mixtures and to analyze proteolytic digests, dot blots were performed using rabbit polyclonal antibodies generated against CobC (Envigo, Indianapolis, IN). For dot blot analysis, 3 μL of 1 mM proteoliposomes obtained by liposome flotation and 100 ng of positive controls was spotted onto a nitrocellulose membrane. Membranes were incubated for 30 min in blocking buffer of phosphate-buffered saline containing Tween 20 (PBST) comprised of NaH_2_PO_4_ (10 mM, pH 7.2), NaCl (0.9% [wt/vol]), Tween 20 (0.1% [vol/vol]), and instant dry milk (5% [wt/vol]). Membranes were probed with anti-CobC or anti-CobS antibodies (1:5,000 in blocking buffer) for 1 h, then washed three times (30 min each) with PBST. Membranes were probed for 1 h with horseradish peroxidase (HRP)-conjugated goat anti-rabbit secondary antibodies (Sigma) in PBST (1:10,000) before three 30-min washes with PBST. Membranes were incubated in SuperSignal West Pico Plus chemiluminescent substrate (Thermo Fisher) for 5 min and imaged using a UVP ChemStudio imaging instrument (Analytik Jena). Purified CobC protein was used as a positive control, and a SuperSignal molecular weight protein ladder (Thermo Fisher) was used as a reference for the electrophoretic behavior of molecules of known molecular masses.

### Microscopy.

The effect of CobS overproduction on the cell membrane was visualized by fluorescence microscopy. Starter cultures of E. coli C41(λDE3) harboring empty cloning vector or plasmid encoding CobS protein were grown for 16 h in LB containing antibiotic at 37°C shaking at 150 rpm. Starter cultures were used to inoculate (10% [vol/vol]) 5 mL of LB containing antibiotic. Cultures were incubated at 37°C with shaking at 150 rpm. Expression of *cobS^+^* was induced by the addition of IPTG to a final concentration of 1 mM when cultures reached an OD_600_ of ∼0.5. After the addition of IPTG, cultures were incubated for an additional 30 min. A 0.5-mL sample of culture was incubated for 5 min with the vital membrane stain FM4-64FX (Thermo Fisher) and 4′,6-diamidino-2-phenylindole (DAPI; Thermo Fisher) at final concentrations of 5 μg mL^−1^ and 2 μg mL^−1^, respectively. Cells were subjected to centrifugation at 6,000 × *g* for 4 min and washed with 0.5 mL of PBS before resuspension in 0.1 mL of PBS. A 1.5-μL sample of stained cell suspension was applied to a 1% (wt/vol) agarose pad for imaging. Images were collected using a Nikon Eclipse Ni microscope equipped with a CoolSNAP MYO camera (Photometrics) using filter sets for DAPI (exciter, ET395/25×, emitter, ET460/50m, and dichroic, T425lpxr) and mCherry/Texas Red long pass (for FM4-64FX, exciter, ET560/40×, emitter, ET590lp, and dichroic, T590lpxr).

### Ethidium bromide accumulation assay.

The effect of CobS overproduction and CobC and CobS coexpression on membrane permeability was examined by a modified ethidium bromide accumulation assay as outlined elsewhere (48). Briefly, starter cultures of E. coli C41(λDE3) harboring empty cloning vector or plasmids encoding CobS^WT^ or CobS^D82A^ alone or both CobC^WT^ and CobS^WT^ proteins were grown overnight in LB containing antibiotic at 37°C with shaking at 150 rpm. Starter cultures were subcultured (1% [vol/vol] inoculum) into 198 μL of LB plus antibiotic in a 96-well microtiter plate (Falcon) and incubated in a plate reader (BioTek EON) at 37°C with orbital shaking. At an OD_630_ of ∼0.4, IPTG was added to a final concentration 0, 0.5, or 1 mM to induce expression of *cobS*^+^ alone or both *cobS*^+^ and *cobC*^+^. Cells were incubated for 30 min as described above. Cultures (150 μL) were transferred into the wells of a black, round-bottom 96-well microtiter plate, and ethidium bromide was added to a final concentration of 6.25 μM. Relative fluorescence (excitation, 530 nm; emission, 600 nm) was monitored immediately upon addition of ethidium bromide using a BioTek Gemini fluorescent plate reader for 180 s. The rate of relative fluorescence (relative fluorescence units [RFU]/s) was determined by linear regression. Significance was determined by unpaired Student's *t* test using Prism version 8 (GraphPad) software.

### Flow cytometry.

The effects of CobS overproduction on membrane potential and permeability were examined using the BacLight membrane potential kit (Invitrogen) containing 3,3′-diethyloxacarbocyanine iodide [DiOC_2_(3)] and TO-PRO-3 dyes (Molecular Probes). These experiments were modified from protocols described elsewhere (49, 50). Starter cultures of E. coli C41(λDE3) harboring empty cloning vector or plasmids encoding CobS^WT^ or CobS^D82A^ proteins were grown for 16 h in LB containing antibiotic at 37°C shaking at 150 rpm. Starter cultures were used to inoculate (10% [vol/vol]) 5 mL of LB containing antibiotic. Cultures were incubated at 37°C with shaking at 150 rpm. Expression of *cobS* alleles was induced by the addition of IPTG to a final concentration of 0.5 or 1 mM when cultures reached an OD_600_ of ∼0.4. After the addition of IPTG, cultures were incubated for an additional 15 or 30 min. The final optical density at 600 nm was determined, 1 × 10^6^ cells/mL were added to 1 mL of PBS, DiOC_2_(3) and TO-PRO-3 were added to final concentrations of 30 μM and 0.5 mM, respectively, and the cultures were incubated for 30 min at 24°C before analyzing the fluorescence using a CyAn ADP instrument (Beckman Coulter). Refer to Fig. S4 in the supplemental material for additional details on flow cytometry analysis. Cells and dyes were detected using forward scatter (FSC), side scatter (SSC), FL1 (488 nm/530 nm), FL3 (488 nm/613 nm), and FL9 (633 nm) channels. Data were analyzed as outlined by the BacLight membrane potential kit. Significance was determined by one-way analysis of variance (ANOVA) with *post hoc* Bonferroni multiple comparison. Cells subjected to carbonyl cyanide 3-chlorophenylhydrazone (CCCP; 5 μM) and polymyxin B (1 μg/mL) were used as controls for DiOC_2_(3) and TO-PRO-3, respectively.

10.1128/mBio.02697-21.4 FIG S4 Flow cytometry analysis. (A) Side scatter (SS log) and forward scatter (FS log) plots are gated to remove debris, yielding population P1, which includes permeabilized and intact cells. (B) TO-PRO-3 fluorescence is used to determine the percentage of the P1 population that is permeabilized. Intact cells are gated to yield P2. Cells treated with polymyxin B (1 μg/mL) were used to determine gating. Mean fluorescence intensity (MFI) of P2 is calculated for both red and green fluorescence of DiOC_2_(3). Cells treated with CCCP (5 μM) were used to determine gating of cells with compromised membrane potential (DY). The ratio of red to green MFI is used to compare the membrane polarization across populations. Download FIG S4, PDF file, 0.7 MB. Copyright © 2022 Jeter and Escalante-Semerena. 2022 Jeter and Escalante-Semerena. https://creativecommons.org/licenses/by/4.0/ This content is distributed under the terms of the Creative Commons Attribution 4.0 International license.

### Cell viability assay.

The effect of CobS^WT^ overproduction and CobC^WT^ and CobS^WT^ coexpression on cell viability was determined by counting CFUs. Cultures were grown, and expression of *cobS*^+^ alone or *cobC*^+^ and *cobS*^+^ was induced as outlined above for flow cytometry. Cells were serially diluted in sterile 0.85% (wt/vol) NaCl, plated on LB containing 1.5% (wt/vol) agar and ampicillin (100 μg mL^−1^) or kanamycin (50 μg mL^−1^), and incubated for 16 h at 37°C before colonies were counted. Cultures that were not induced were normalized to 1 and compared to cultures expressing CobS^WT^ or CobC^WT^ and CobS^WT^ as a ratio of CFUs. The assay was performed in biological triplicate with three technical replicates each time. Significance was determined by unpaired Student's *t* test using Prism (GraphPad) v8 software.

### Data availability.

All the data generated in this study are included in the article.
