# Supplementary figures and images for: Elevated Levels of an Enzyme Involved in Coenzyme B12 Biosynthesis Kills Escherichia coli
Source: mBio. 2022 Jan 11;13(1):e02697-21. doi: 10.1128/mbio.02697-21 (PMC8749415; doi:10.1128/mbio.02697-21)

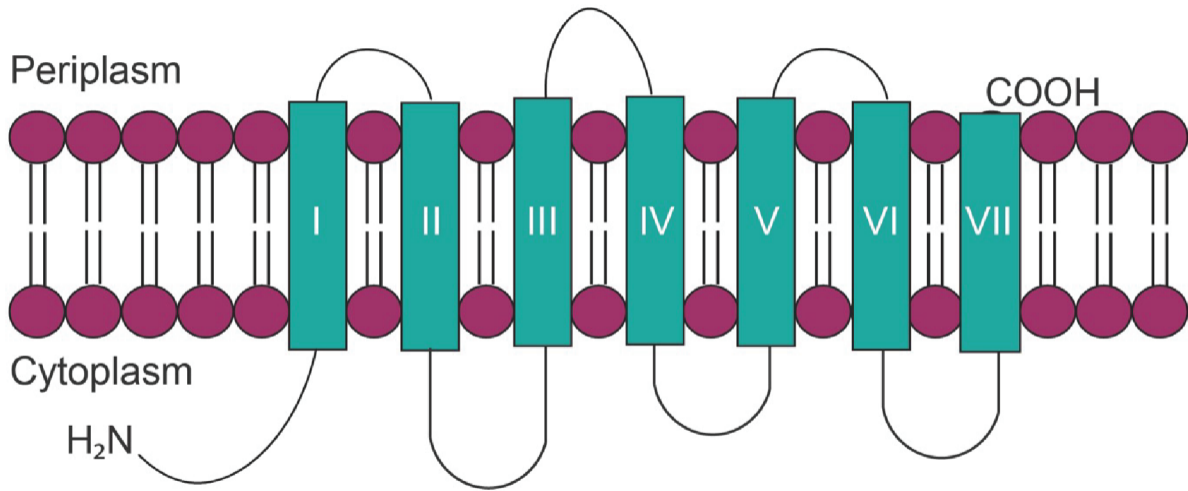

Supplement: FIG S1 [file mbio.02697-21-sf001.pdf]

A

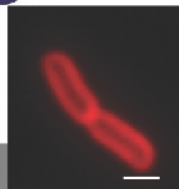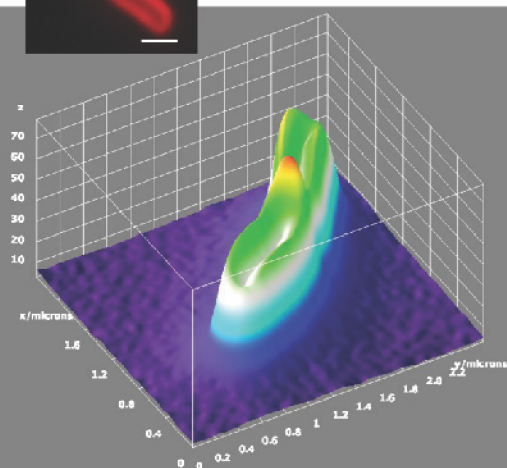

B

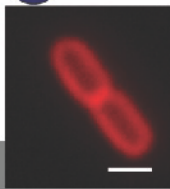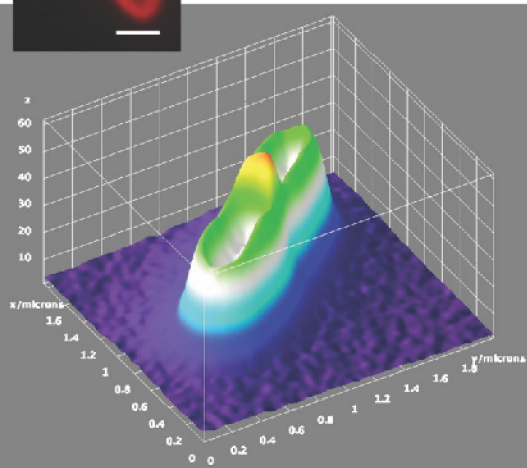

C

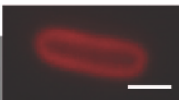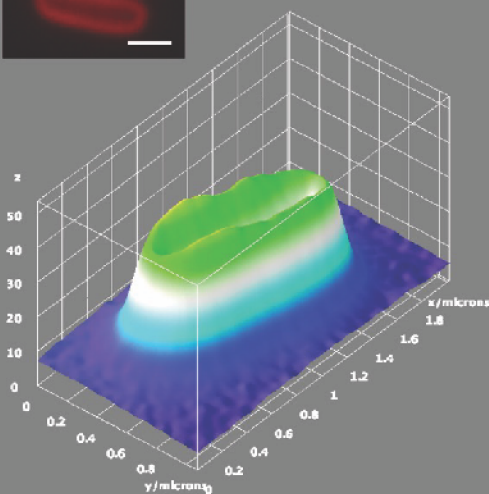

D

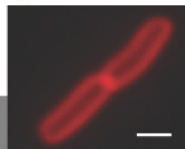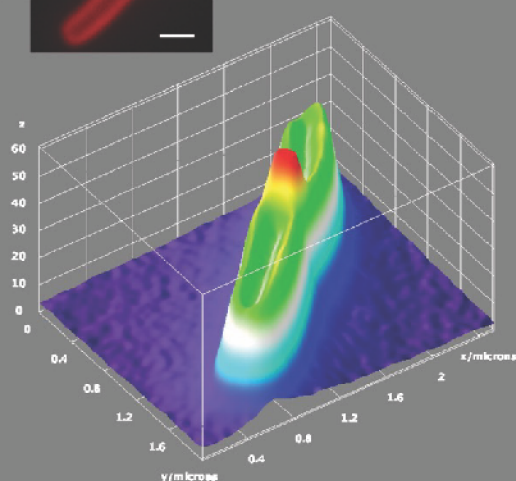

Supplement: FIG S5 [file mbio.02697-21-sf005.pdf]

**A**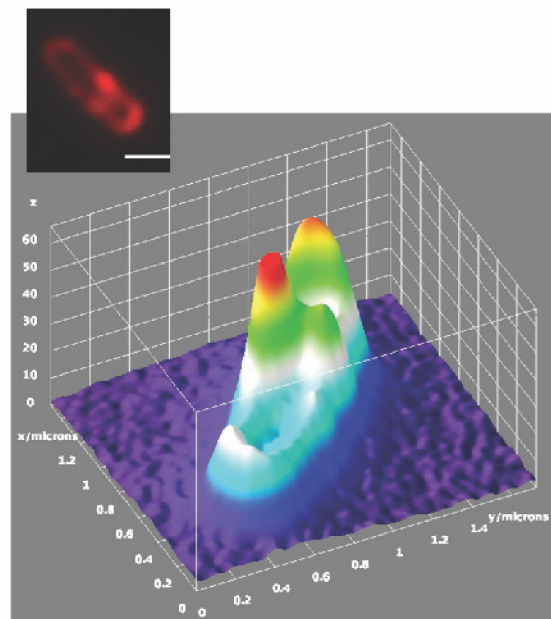**B**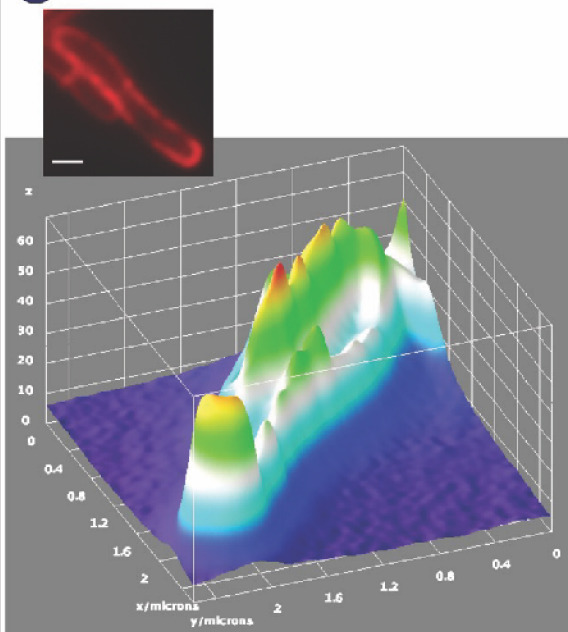**C**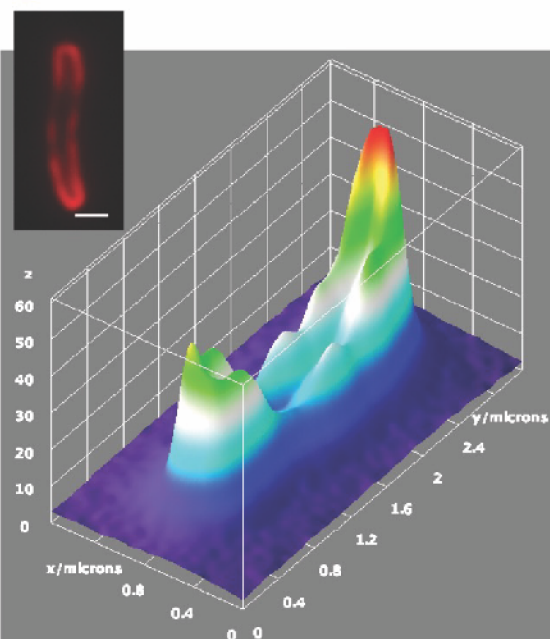**D**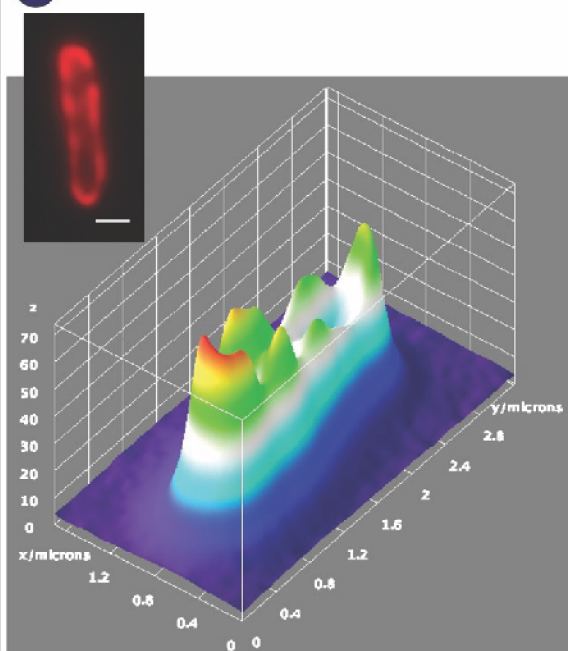

Supplement: FIG S6 [file mbio.02697-21-sf006.pdf]

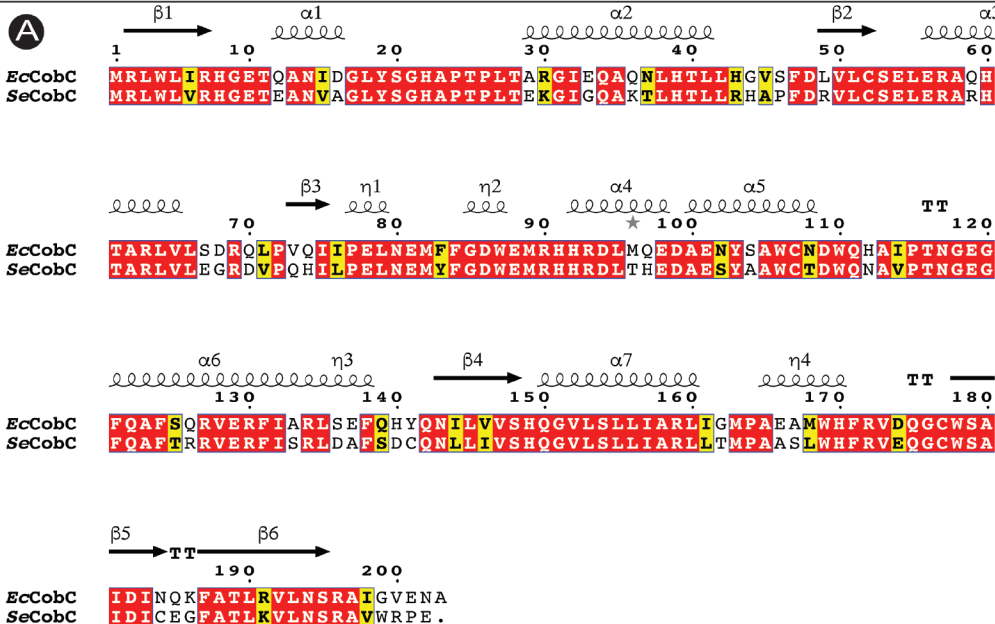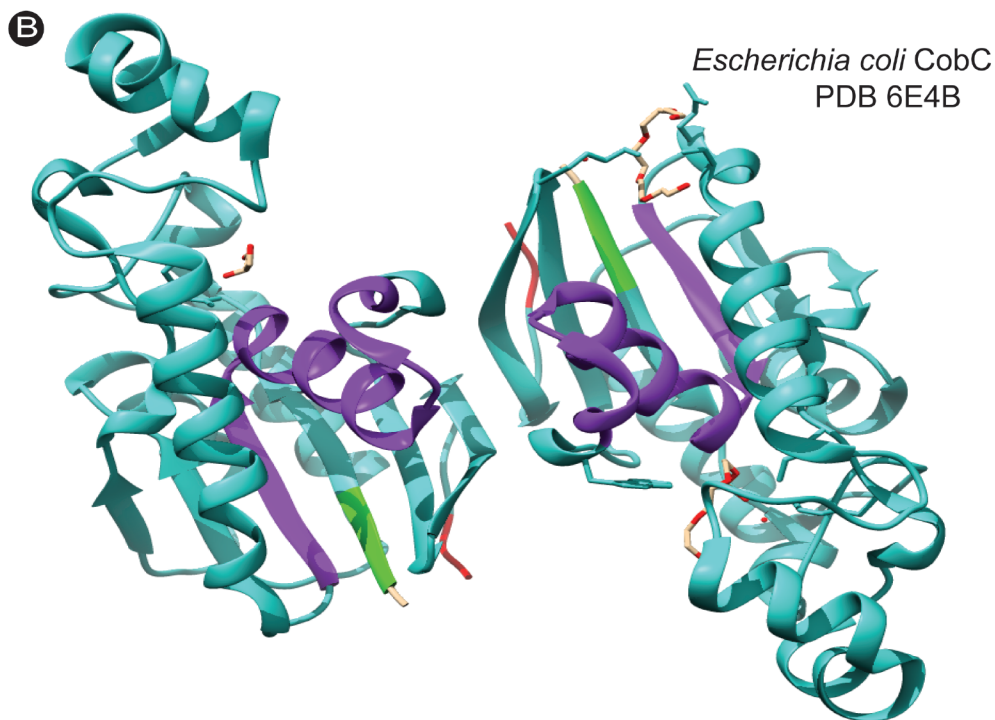

Supplement: FIG S7 [file mbio.02697-21-sf007.pdf]

A

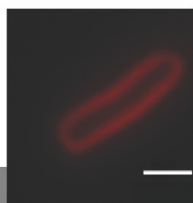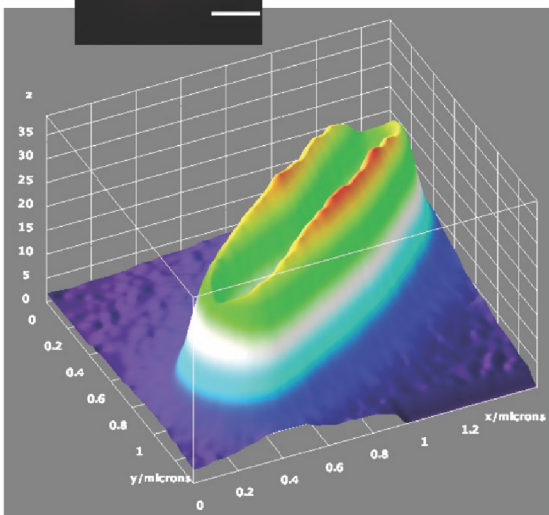

B

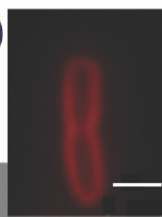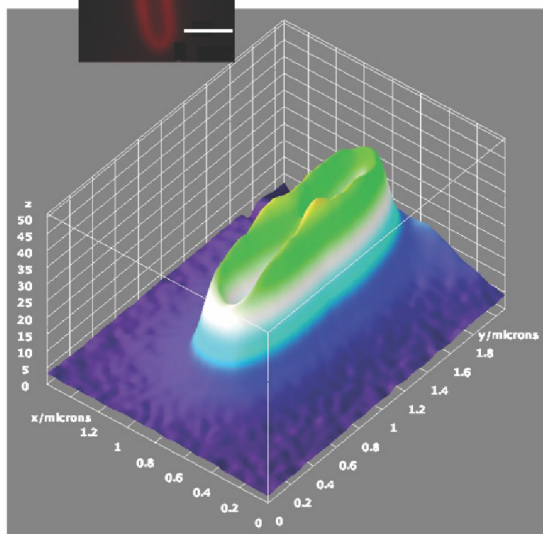

C

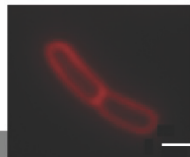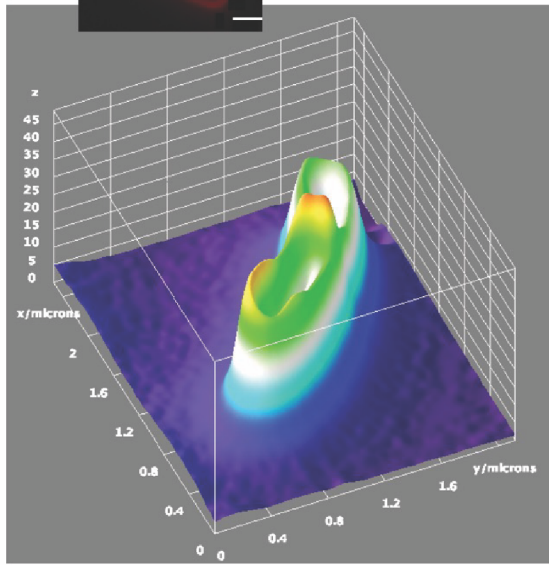

D

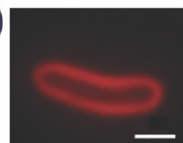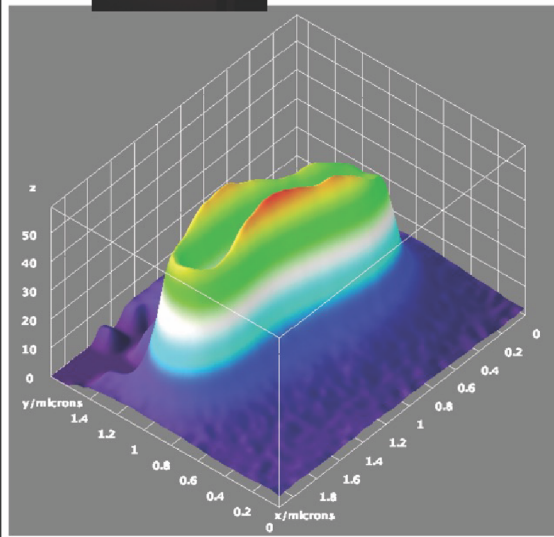

Supplement: FIG S8 [file mbio.02697-21-sf008.pdf]

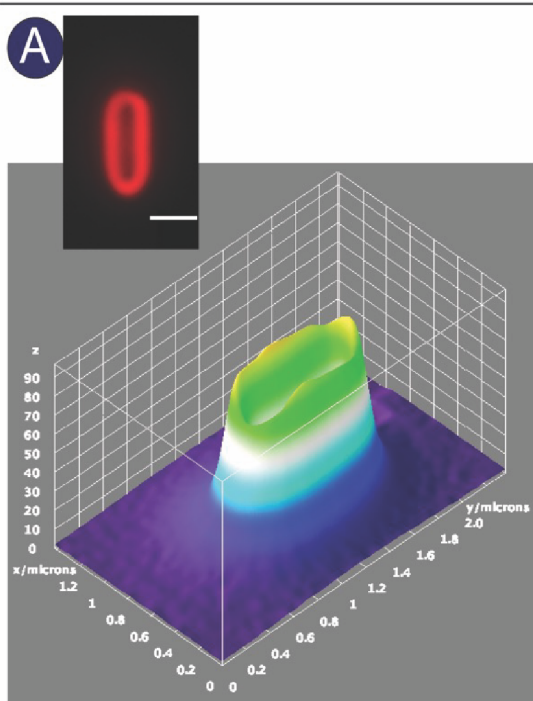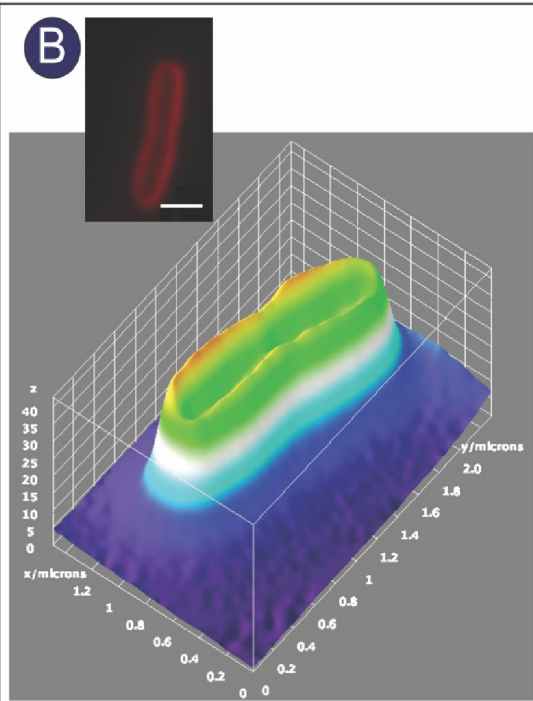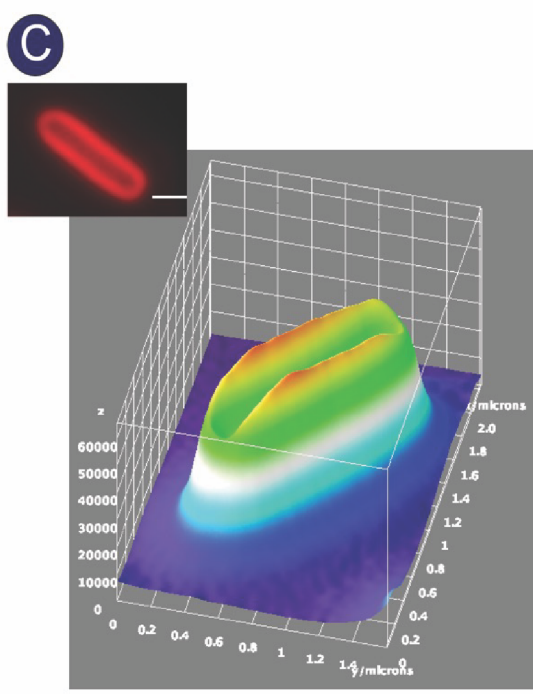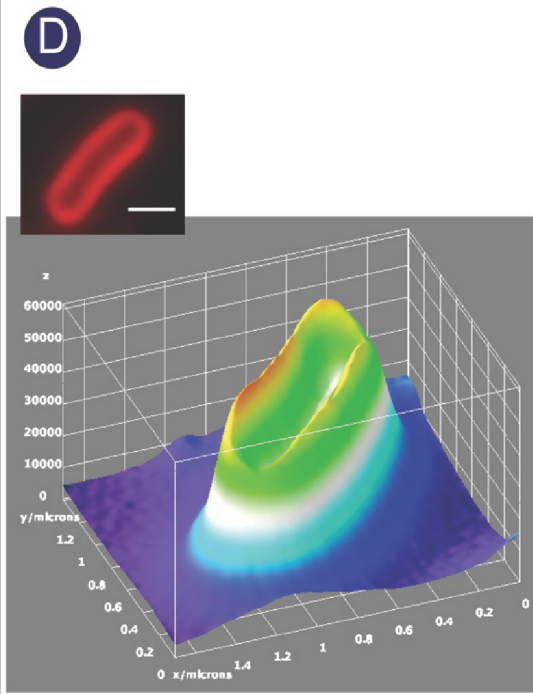

Supplement: FIG S9 [file mbio.02697-21-sf009.pdf]

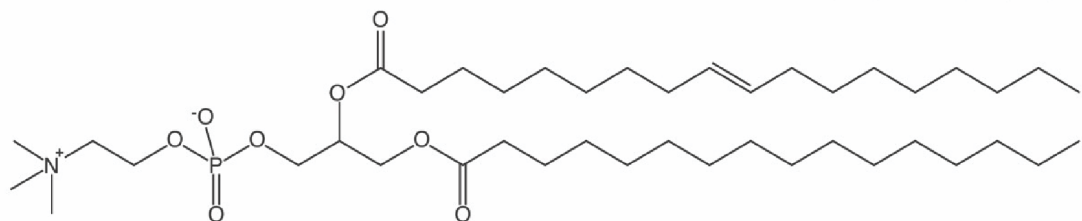

80% **POPC**

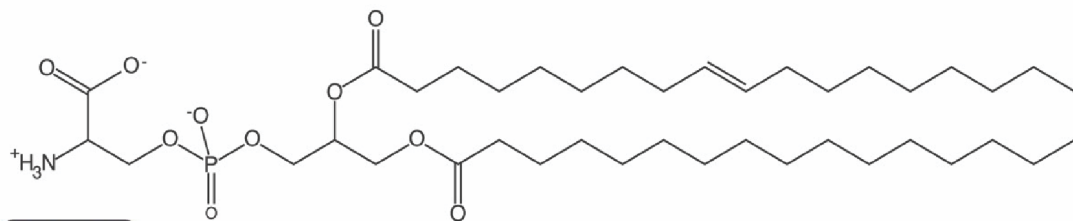

10% **POPS**

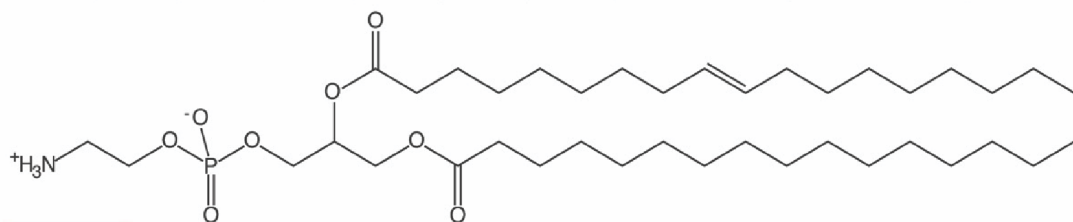

9.5% **POPE**

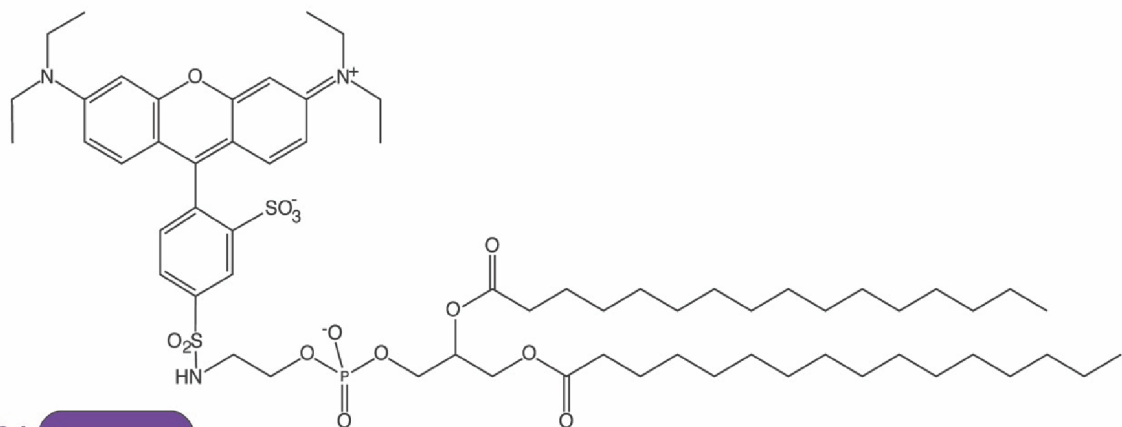

0.5% **Rh-DHPE**

Supplement: FIG S3 [file mbio.02697-21-sf003.pdf]

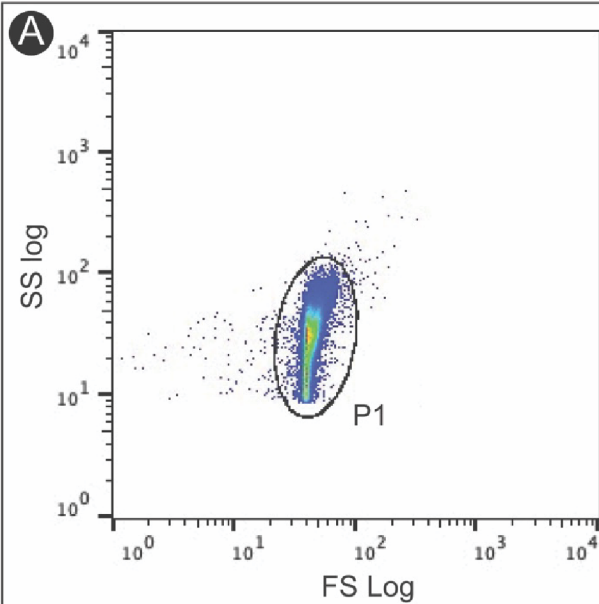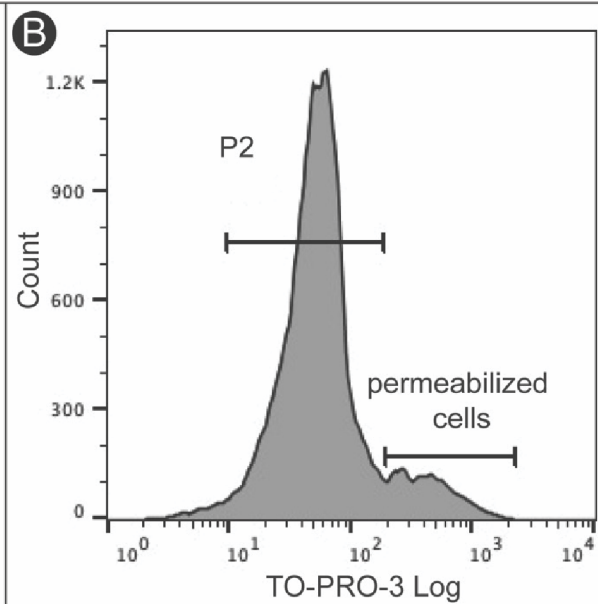

Supplement: FIG S4 [file mbio.02697-21-sf004.pdf]
