# Supplementary material for: Elevated Levels of an Enzyme Involved in Coenzyme B12 Biosynthesis Kills Escherichia coli
Source: mBio. 2022 Jan 11;13(1):e02697-21. doi: 10.1128/mbio.02697-21 (PMC8749415; doi:10.1128/mbio.02697-21)
Supplement: FIG S2 [file mbio.02697-21-sf002.pdf]

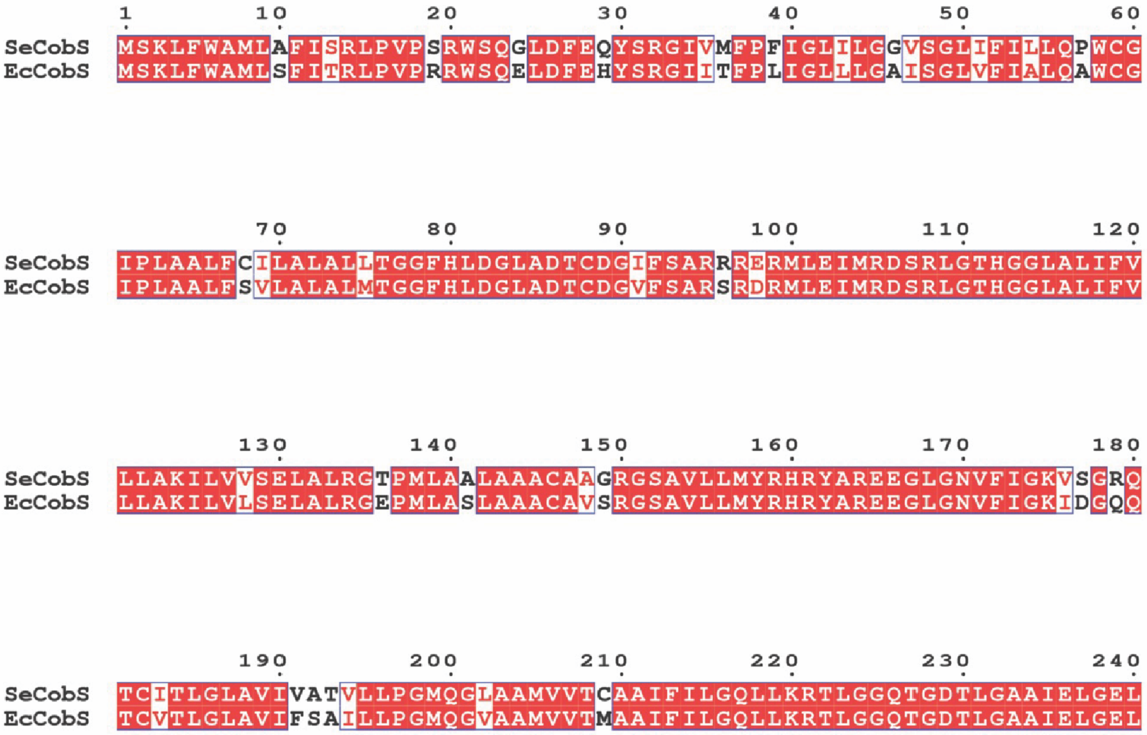
1 10 20 30 40 50 60
SeCobS MSKLFWAMLAFISRLPVPSRWSQGLDFEQYSRGIVMFPFIGLILGGVSGLIFILLQPWCG
EcCobS MSKLFWAMLSFITRLPVPRRWSQELDFEHYSRGIITFPLIGLLLGAISGLVFIALQAWCG
70 80 90 100 110 120
SeCobS IPLAALFCILALALLTGGFHLDGLADTCDGIFSARRRERMLEIMRDSRLGTHGGLALIFV
EcCobS IPLAALFSVLALALMTGGFHLDGLADTCDGVFSARSRDRMLEIMRDSRLGTHGGLALIFV
130 140 150 160 170 180
SeCobS LLAKILVVSELALRGTPMLAALAAACAAGRGSAVLLMYRHRYAREEGLGNVFIGKVSGRQ
EcCobS LLAKILVLSELALRGEPMLASLAAACAVSRGSAVLLMYRHRYAREEGLGNVFIGKIDGQQ
190 200 210 220 230 240
SeCobS TCITLGLAVIVATVLLPGMQGLAAMVVTCAAIFILGQLLKRTLGGQTGDTLGAAIELGEL
EcCobS TCVTLGLAVIFSAILLPGMQGVAAMVVTMAAIFILGQLLKRTLGGQTGDTLGAAIELGEL
